# Location and amount of joint involvement differentiates rheumatoid arthritis into different clinical subsets

**DOI:** 10.1038/s41746-025-01997-1

**Published:** 2025-10-23

**Authors:** Tjardo D. Maarseveen, Marc P. Maurits, Lavinia Agra Coletto, Simone Perniola, Stefan Böhringer, Nils Steinz, Sytske Anne Bergstra, Dario Bruno, Maria Rita Gigante, Viviana A. Pacucci, Luca Petricca, Bianca Boxma-de Klerk, Herman Kasper Glas, Clara Di Mario, Denise Campobasso, Barbara Tolusso, Josien Veris-van Dieren, Annette H. M. van der Helm-van Mil, Elisa Gremese, Maria Antonietta D’Agostino, Marcel J. T. Reinders, Marco Gessi, Tom W. J. Huizinga, Stefano Alivernini, Erik B. van den Akker, Rachel Knevel

**Affiliations:** 1 Department of Rheumatology, Leiden University Medical Center, Leiden, Netherlands; 2 Division of Rheumatology – Fondazione Policlinico Universitario A. Gemelli IRCCS, Rome, Italy; 3 Division of Clinical Immunology - Fondazione Policlinico Universitario A. Gemelli IRCCS, Rome, Italy; 4 Department of Medical Statistics and Bioinformatics, Leiden University Medical Center, Leiden, Zuid-Holland Netherlands; 5 Rheumatology outpatient clinics, Reumazorg Zuid West Nederland, Goes, Netherlands; 6 Immunology Research Core Facility – Gemelli Science and Technology Park (GSTeP) - Fondazione Policlinico Universitario A. Gemelli IRCCS, Rome, Italy; 7 Department of Rheumatology, Erasmus Medical Center, Rotterdam, Netherlands; 8 Università Cattolica del Sacro Cuore, Rome, Italy; 9 Leiden Computational Biology Centre, Leiden University Medical Center, Leiden, Zuid-Holland Netherlands; 10 Delft Bioinformatics Lab, Delft University of Technology, Delft, Zuid-Holland Netherlands; 11 Institute of Pathology - Fondazione Policlinico Universitario A. Gemelli IRCCS, Rome, Italy; 12 Rheumatology, Newcastle University Translational and Clinical Research Institute, Newcastle upon Tyne, United Kingdom

**Keywords:** Rheumatoid arthritis, Classification and taxonomy, Computational models, Data integration, Data processing, Machine learning, Bioinformatics, Inflammation, Computational biology and bioinformatics, Immunology, Medical research

## Abstract

Rheumatoid arthritis (RA) is a heterogeneous disease with variable symptoms, prognosis, and treatment response, necessitating refined patient classification. We applied multimodal deep learning and clustering to identify distinct RA phenotypes using baseline clinical data from 1,387 patients in the Leiden Rheumatology clinic. Four Joint Involvement Patterns (JIP) emerged: foot-predominant arthritis, seropositive oligoarticular disease, seronegative hand arthritis, and polyarthritis. Findings were validated in clinical trial data (*n* = 307) and an independent secondary care cohort (*n* = 515). Clusters showed high stability and significant differences in remission rates (*P* = 0.007) and methotrexate failure (*P* < 0.001). JIP-hand patients had superior outcomes (particularly in ACPA-positive patients) versus JIP-foot (HR:0.37, *P* < 0.001) and JIP-poly (HR:0.33, *P* = 0.005), independent of baseline disease activity and clinical markers. Synovial histology analysis (*n* = 194) revealed distinct inflammatory patterns across clusters, hinting at different underlying biological mechanisms. These validated RA phenotypes based on joint involvement patterns may enable targeted research into disease mechanisms and personalized treatment strategies.

## Introduction

Rheumatoid arthritis (RA) is a heterogeneous disease. The current classification criteria for RA were developed to approximate the decision to start early treatment and exclude other diseases. At clinical presentation, patients vary in the number and pattern of joints involved, presence of extra-articular manifestations, and abnormalities in blood and synovial tissue^1–3^. The heterogeneity of RA also manifests in clinical outcomes, namely prognosis, treatment response, and comorbidities. This evident diversity likely impacts the interpretation of treatment effects and etiologic factors such as genetics, and may downplay their importance altogether^4^. If phenotypic subsetting into more homogeneous groups is possible, it could improve research into the etiology of RA and enhance its treatment.

For centuries, pattern recognition on clinical variables by doctors has been the driving force of disease identification and examination of the underlying etiologic mechanisms. Thus far, clinicians have not identified the relevant (sub)patterns in RA. The presence of ACPA^5–8^ and the age of onset^9,10^ have been raised as possible dichotomous disease subsetting features. However, neither of these markers in isolation adequately addresses the heterogeneity and complexity of the disease. This suggests that other factors are involved.

Cluster analysis combining a high number of factors has demonstrated its effectiveness in categorizing complex diseases (such as type II diabetes, asthma, and osteoarthritis) into subtypes that differ in clinical outcomes or biological background^11–13^. In the context of RA, there is considerable focus on molecular phenotyping, such as that done by Lewis et al.^14^, who discovered patterns in synovial tissue at baseline, with the lymphoid-myeloid pathotype being a predictor of poor outcome at disease onset^15^. Others used clinical and comorbidity information for clustering and identified four subsets, including one that exhibited a higher likelihood of biological DMARD initiation^16^. Likewise, Curtis et al.^17^ used clinical variables, though not exclusively at baseline, and identified five clusters that differed in disease activity, RA duration, and type of comorbidities. These outcomes are typically highly influenced by treatment decisions and events that occur independently of the specific RA type. Furthermore, detailed clinical information such as the pattern of involved joints may be relevant for disease differentiation, as exemplified by psoriatic arthritis (PsA)^18^; yet none of the previous studies capitalize on this information for clustering.

Electronic Health Records (EHR) data provide a powerful asset for clustering as they encompass a wide variety of data modalities (laboratory values, clinical examinations, demographics) that each offer a unique perspective on the patient’s condition. EHRs are collected during routine clinical care and thus resemble the true patient population more closely than study populations collected with a particular hypothesis in mind. The diversity of data types, however, poses a methodological challenge due to structural differences between the data modalities. The recent surge of deep learning tools^19^ offers the possibility of combining different EHR layers into a patient representation by extracting the (hidden) factors that capture the most variation in the data. At present, many machine learning (ML) techniques exist to learn the relevant (clinical) patterns and encode patients accordingly. These embeddings can be used to detect patient subgroups, identify patterns, build predictive models, or assist in making disease classifications. The literature reports that clustering on top of these embeddings typically outperforms conventional techniques in cases of high-dimensional or complicated data^20–22^.

In this study, we aimed to dissect the clinical heterogeneity of RA by using the symptoms at initial presentation, before external factors such as treatment interfere. We hypothesize that the location of the involved joints and the inflammatory patterns observed in the blood play a role in subsetting RA, similar to their significance in distinguishing PsA from RA^18^. To achieve this, we make use of advanced data-driven techniques to identify and analyze disease-differentiating signatures based on initial clinical variables and determine whether they relate to clinical outcomes and histological synovial features.

## Results

### Patients

**Fig. 1:**
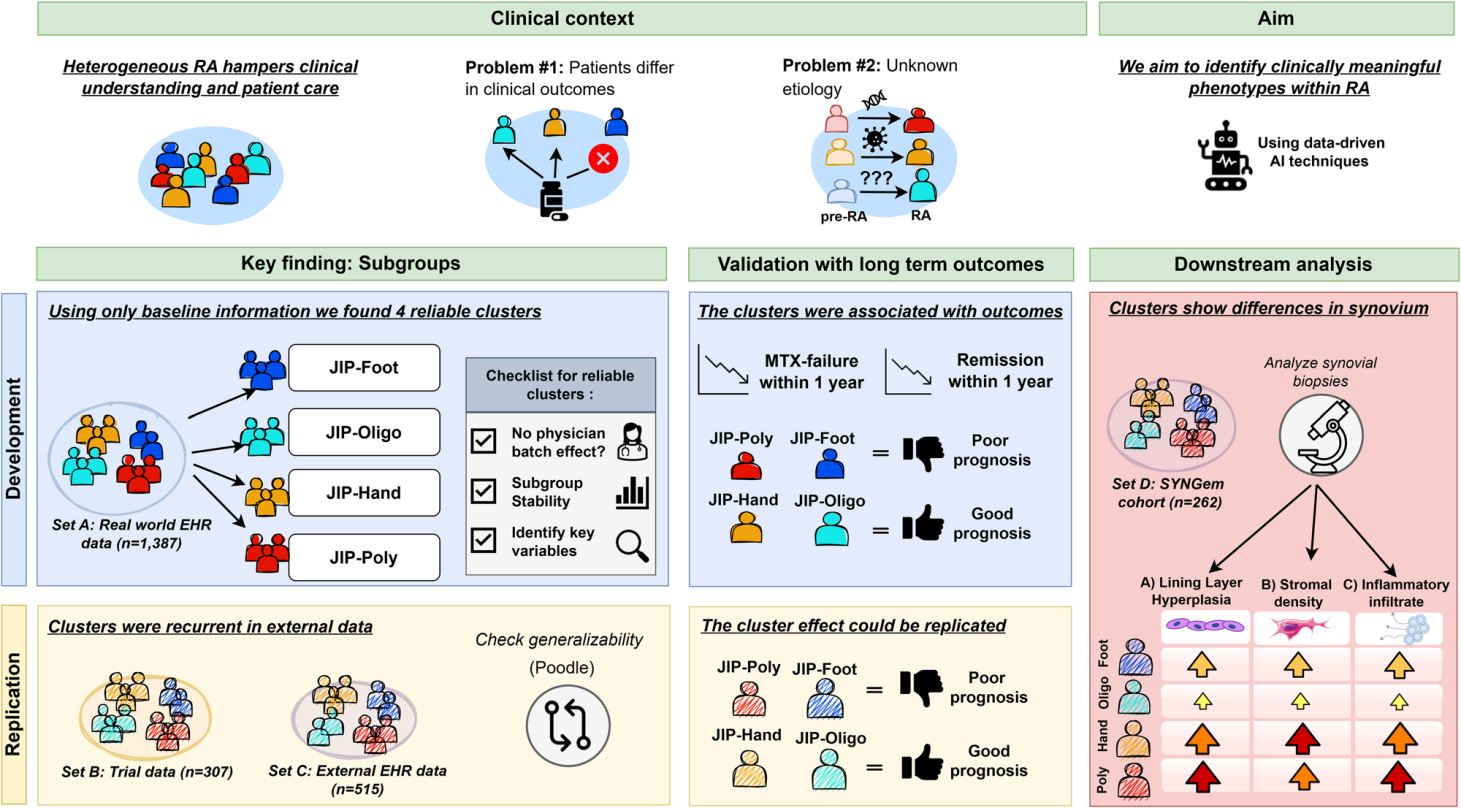
Workflow illustrating the study outline. This study used data-driven analysis to clarify the complexity of rheumatoid arthritis, providing insights into disease etiology and predicting clinical outcomes. Cluster analysis identified four distinct baseline subgroups that differed in MTX treatment response and 1 year remission rates. These clusters and treatment effects were replicated across multiple centers. Additional analysis of a separate cohort showed these clusters also corresponded to synovial differences. The fibroblast vector is from Servier Medical Art (https://smart.servier.com/), licensed under CC BY 4.0.

We retrieved 2691 RA patients for training set A of whom, 1387 were included in our study based on the availability of lab values and joint counts. For the replication, set B and set C had 364 and 1227 RA cases, of whom 307 and 515 had complete information (Supplementary Table 1, Supplementary Fig. 1). For the downstream analysis, we looked at biopsy data from 264 patients of set D, of whom 194 patients had synovial tissue taken from the same joint (Supplementary Table 2). A workflow diagram for the different phases of the study is shown in Fig. 1.

Each dataset captured a typical early RA population^23,24^. In comparison to set A, patients in the replication sets exhibited higher rates of seropositivity and had fewer tender joints. On average, set B patients were younger and set C patients had less inflammation, with a median ESR of 16. We used the phenotypic variables (see methods) of set A to construct the patient embedding. We visualized how much each type of EHR data contributed to our final embedding using a flameplot analysis (Supplementary Fig. 2).

### Four clusters separated by joint location, serology and blood values

The patient embedding showed four distinct clusters (Supplementary Fig. 3 & 4) which were not determined by any single clinical variable, as indicated by the wide dispersion of values. The primary variation across the clusters was their differences in affected joints, leading us to name them Joint Involvement Patterns (JIP). Additionally, the clusters differed in levels of inflammation, age, and seropositivity (Supplementary Table 3, Supplementary Fig. 5):

- Cluster 1: JIP-foot
  moderate number of involved joints, particularly feet joints, younger patients, low leukocyte and thrombocyte levels.
- Cluster 2: JIP-oligo
  limited joint involvement and mostly seropositive patients.
- Cluster 3: JIP-hand
  elderly patients, symmetrical polyarthritis of hands, seronegative.
- Cluster 4: JIP-polyarthritis
  majority seronegative polyarthritis in hand and feet though with lower ESR.

**Fig. 2:**
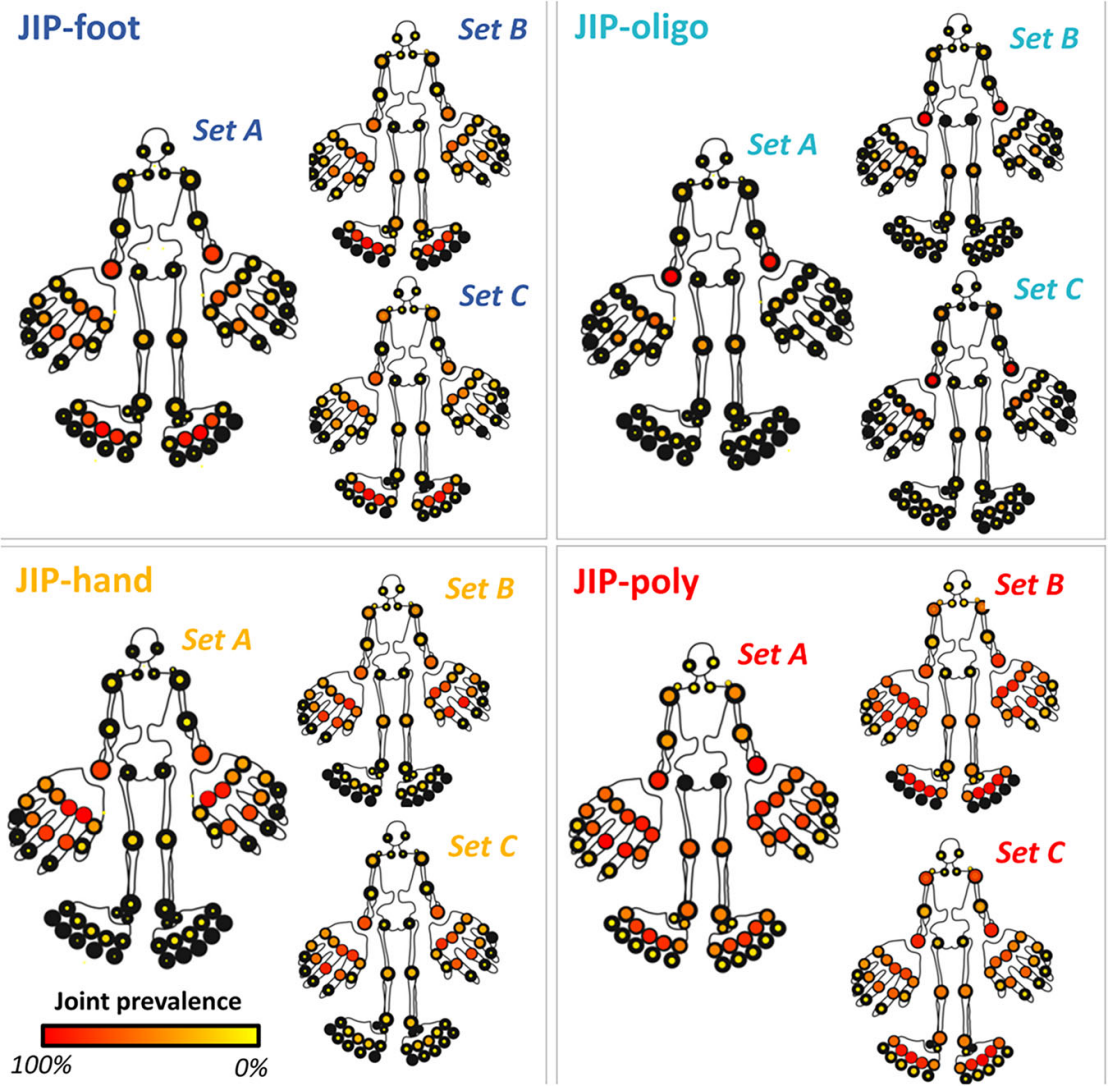
Pictorial mannequins for replication set B and C and their original counterpart (set A) to show the affected joints for each cluster with color and size to depict prevalence. Frequency is colored on a gradient from red (=100%) to yellow (=0%). When there is no colored dot, it signifies the absence of both swelling and pain at baseline for these patients.

The clusters were stable, with an average of >80% of patients grouping together in the same cluster over the 1000 iterations in the stability analysis (Supplementary Figs. 6 and 7). In fact, the stability was better in our combined multimodal approach than if we take each data type (numeric/categorical) separately (Supplementary Fig. 8). The clusters were not driven by treating physicians (Supplementary Fig. 9) and were generalizable across different validation sets (Table 1, Supplementary Fig. 10 & 11), showing similar joint involvement patterns (Fig. 2). Also, the identified patient clusters did not seem to represent different disease stages, as the cluster with the longest symptom duration had the lowest joint count and vice versa. There were differences in cluster prevalences between the validation sets (Supplementary Table 4 and 5).

**Table 1:** Baseline characteristics of the different patient clusters (set A + replication sets B & C)

	JIP-Foot	JIP-Oligo	JIP-Hand	JIP-Poly
N	596	761	450	402
Sex, female ^ɣ^ [n(%)]	389 (65.3)	505 (66.4)	272 (60.4)	272 (67.7)
Age ^ɣ^ (SD, yr)	56.6 (14.6)	59.5 (14.6)	66.4 (13.2)	54.7 (14.7)
RF ^ɣ^ [n(%)]	370 (62.1)	497 (65.3)	200 (44.4)	196 (48.8)
ACPA ^ɣ^ [n(%)]	355 (59.6)	449 (59.0)	162 (36.0)	183 (45.5)
ESR ^ɣ^ (IQR, mm/hr)	22 (9–36)	24 (11–38)	28 (13-48)	22 (9–40)
DAS44(3) (IQR)	3.5 (3.0–4.0)	2.4 (1.9–2.8)	3.7 (3.2–4.3)	4.6 (4.0–5.5)
SJC (IQR)	8 (5–12)	2 (1–5)	10 (7−15)	15 (9−22)
TJC (IQR)	11 (8–15)	3 (2−5)	11 (7−15)	22 (15−30)
DAS28(3) (IQR)	5.2 (4.5–6.0)	4.0 (3.3–4.7)	5.5 (4.9−6.2)	6.5 (5.5–7.2)
Follow up (IQR, days)	1307 (733–2020)	1428 (760–2082)	1127 (566–1875)	1512 (1012-2246)
Symptom duration * (IQR, days)	143 (56–364)	186 (70–399)	101 (48–279)	147 (56-357)

Where *SD* standard deviation, *RF* rheumatoid factor, *ACPA* anti-cyclic citrullinated peptide antibodies, *ESR* erythrocyte sedimentation rate, *IQR* interquartile range, *DAS* three component disease activity score (either 44 or 28 joint scheme), *SJC* swollen joint count, *TJC* tender joint count, ɣ clinical variables that were used for clustering (set A), *symptom duration was only calculated for patients from set A and B.

#### Validation on clinical outcomes beyond baseline

**Fig. 3:**
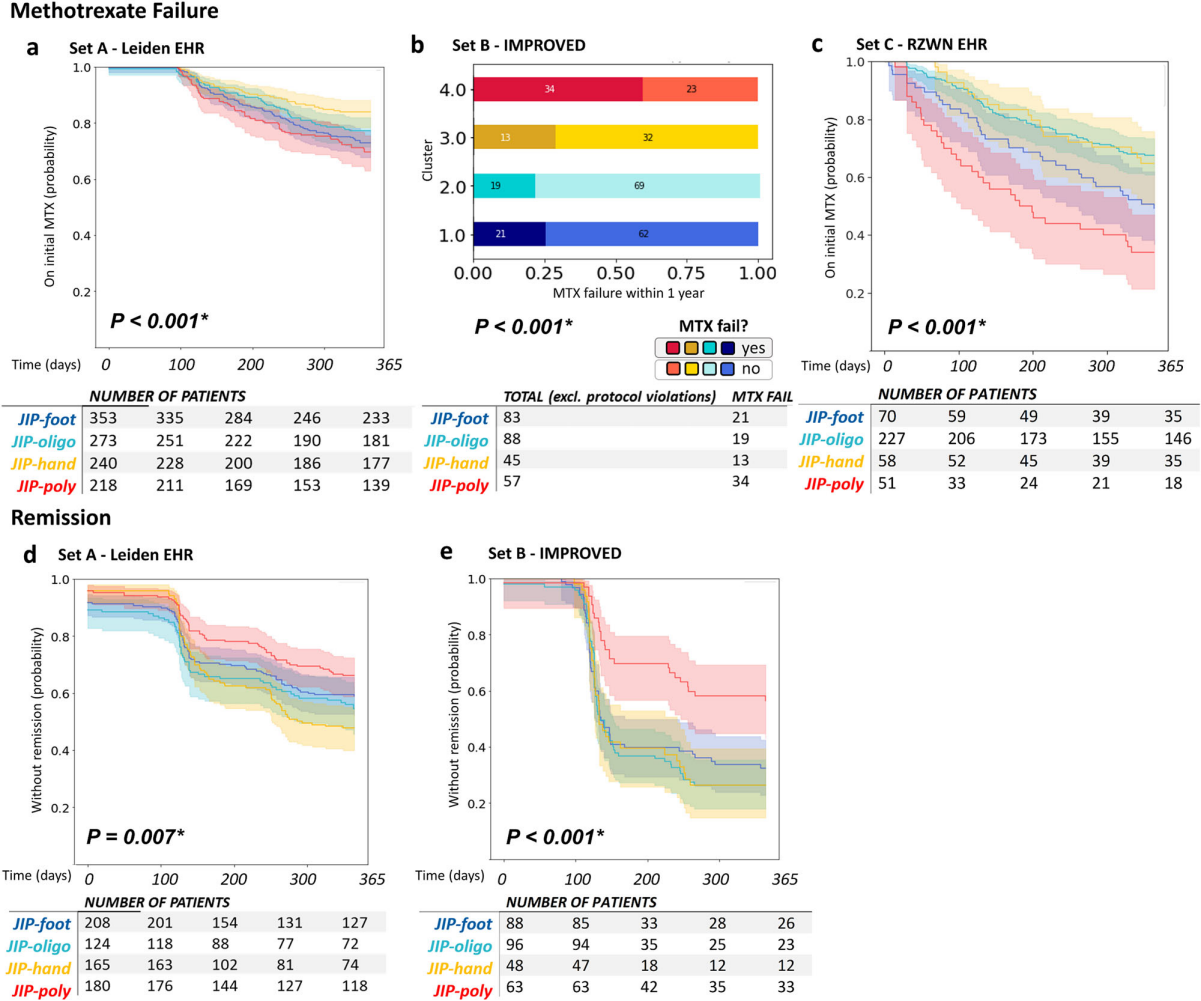
Association of baseline joint involvement clusters with clinical outcomes at 1 year. **a** Kaplan–Meier curves for MTX switch rates in set A MTX-starters (*n* = 1,084). **b** MTX switch rates in set B (*n* = 273). **c** Kaplan-Meier curves for MTX switch rates in set C (*n* = 406). **d** Remission rates (DAS44 < 1.6) in set A (*n* = 677). **e** Remission rates in set B (*n* = 295). Survival curves were generated for time-to-event data (sets A and C), while cross-tabulations were used for set B, where MTX switching occurred at a fixed time per study protocol. Statistical comparisons were performed using log-rank tests for survival curves and chi-squared tests for cross-tabulated data.

In set A, 80% of patients received MTX as an initial drug across all clusters. The Kaplan-Meier curves show a difference in MTX failure between the clusters: 27%, 23%, 16%, 30% (for clusters 1–4, *P* < 0.001, Fig. 3a). The JIP-hand had clearly the best prognosis, where patients were twice as likely to stay on MTX compared to the most severe disease subtype, JIP-poly (HR: 0.48 (95% CI 0.35-0.77), *P* < 0.001). Additionally, the JIP-hand performed better than the JIP-foot (HR: 0.55 (0.37–0.82), *P* = 0.003).

Consistent with MTX response, we observed differences in remission rates: 44.3%, 47.4%, 55.7%, 38.5% (for clusters 1–4, *P* = 0.007, Fig. 3b), with the biggest difference between the JIP-hand and JIP-poly (HR. 1.65 (95% CI 1.2–2.29), *P* = 0.002), even when corrected for baseline disease activity (Supplementary Fig. 12).

#### ACPA within the clusters

**Fig. 4:**
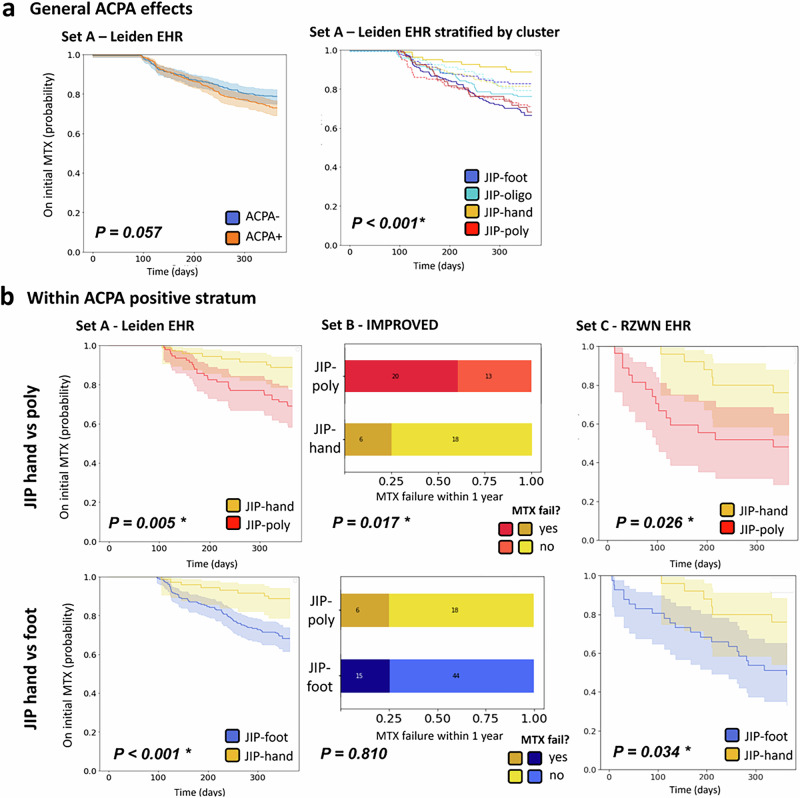
Methotrexate switch rates compared across ACPA status and joint involvement patterns within the seropositive stratum. **a** Kaplan–Meier curves showing MTX switch rates by ACPA status overall and stratified by cluster, where dashed lines represent ACPA-positive patients. **b** Comparison of MTX switch rates between JIP-hand and foot clusters (JIP-foot and JIP-poly) within ACPA-positive patients. Statistical comparisons used log-rank tests for survival curves and chi-squared tests for categorical data.

Since the literature reports that ACPA is indicative of persistent disease^25^, we examined whether the ACPA status was the main factor driving the difference in MTX failure. In set A, we found a higher treatment failure in ACPA-positive than ACPA-negative patients (27.6% versus 22.0%, Fig. 4), though it was not significant (*P* = 0.057). Moreover, the association of ACPA with MTX failure differed within the clusters (*P* < 0.001, Fig. 4).

The difference between JIP-hand and the foot clusters JIP-poly and JIP-foot was larger within the ACPA-positive stratum (JIP-hand versus JIP-foot: HR 0.37 (0.15-0.60), *P* < 0.001; JIP-hand vs JIP-poly: HR 0.33 (0.15-0.72), *P* = 0.005) (Fig. 4b). For remission, we could not find this difference in the ACPA-positive stratum.

The good response in JIP-hand raised the question of whether this group overrepresented patients with parvovirus-induced arthritis instead of RA, but none of our clusters were enriched for parvovirus-positive patients (Supplementary Fig. 13).

#### Replication

In set C, 79% of patients received MTX as the initial drug across all clusters, whereas in set B, all patients were administered MTX. In both replication sets, we again observed superior outcomes for the JIP-hand cluster regarding MTX failure (global *P* < 0.001 and *P* < 0.001). Remission could only be tested in replication set B, where it confirmed our previous finding (*P* < 0.001). Consistent with the original finding, the difference between JIP-hand and JIP-poly was particularly pronounced within the ACPA-positive stratum in both replication set B (OR: 0.22 (0.12-0.63), *P* = 0.017) and set C (HR: 0.38 (0.22-0.68), *P* < 0.001). Within the ACPA-positive stratum, we also found a significant difference between JIP-hand and JIP-foot in replication set C (HR: 0.37 (0.15-0.93), *P* = 0.034), though not in replication set B (OR: 1.03 (0.34-3.05), *P* = 0.801). All analyses remained significant when corrected for baseline DAS (Supplementary Fig. 14). Even after adjusting for symptom duration in sets A and B, the association between cluster and treatment persisted (Supplementary Fig. 15).

#### Informative value of clusters beyond known risk factors for MTX failure

To ensure that the association between clusters and treatment outcomes was not solely driven by established clinical markers, we adjusted the Cox regression model accordingly (Supplementary Fig. 16). Notably, the inclusion of well-known contributing factors such as ACPA, RF, and the number of affected joints did not reduce the additional predictive value of clustering (*P* = 0.020 in Set A; *P* = 0.019 in Set C).

#### Diversity in synovial characteristics

Next, we examined histological variations between patient clusters in set D according to the Krenn synovitis components^26^. Biopsies were taken from the most inflamed joint, typically the knee or wrist. Due to the limited number of samples from other joints, a thorough comparison across different anatomical locations was not feasible. Therefore, we primarily focused on a single, consistent location that was involved in most clusters—specifically, the knee joints.

**Fig. 5:**
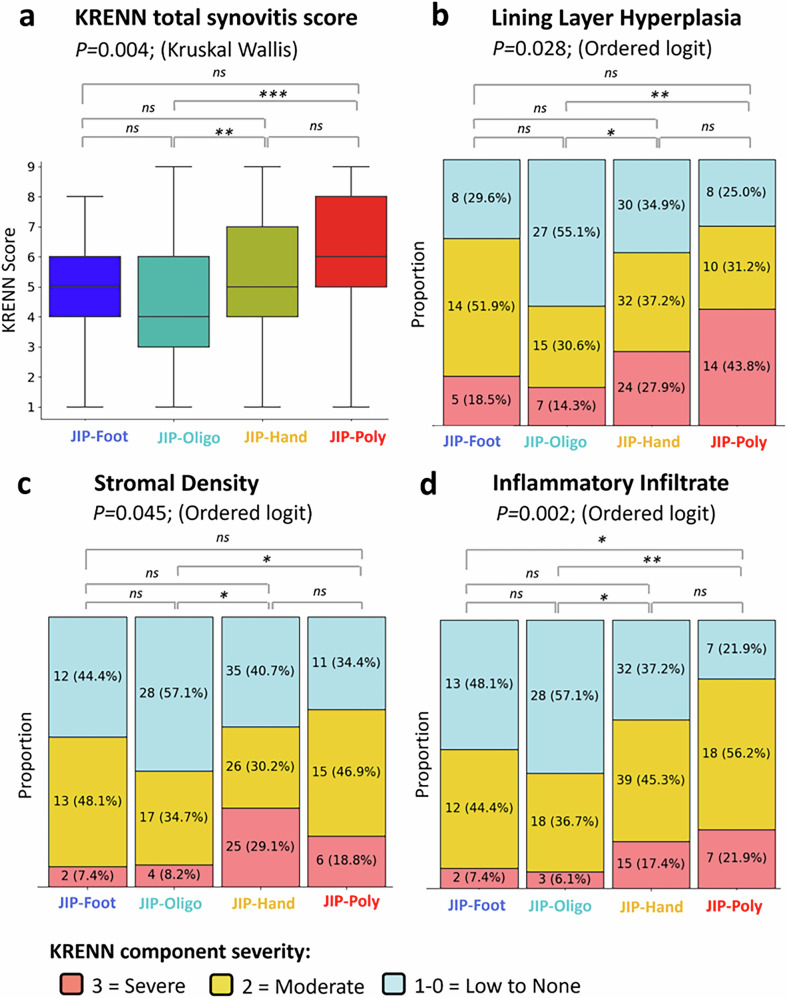
Synovitis severity across the patient clusters. **a** Total Krenn synovitis scores for each cluster. **b** Lining layer hyperplasia scores by cluster. **c** Stromal density scores by cluster. **d** Inflammatory infiltrate scores by cluster. Global trends were assessed using the Kruskal-Wallis test for total Krenn scores and ordered logit models for individual component grades, followed by post hoc Wald tests. Significance levels: *p < 0.05, **p < 0.01, ***p < 0.001.

In total, we analyzed synovial tissue from 194 patients, distributed across four clusters: 27 in JIP-foot, 49 in JIP-oligo, 86 in JIP-hand, and 32 in JIP-poly (see Supplementary Fig. 17). The Krenn synovitis score differed significantly among the clusters, with mean scores of 5, 4, 5, and 6, respectively (*P* = 0.004). The highest synovitis score was observed in the JIP-poly cluster, while the lowest was seen in the JIP-oligo cluster (Fig. 5).

The clusters showed significant histopathological variations between the JIP variants in lining layer hyperplasia (*P* = 0.026), stromal density (*P* = 0.045), and inflammatory infiltrate (*P* = 0.002). These variations were primarily driven by the distinction between the more stereotypical RA clusters (JIP-hand/JIP-poly) and JIP-oligo, which was characterized by low-grade synovitis. In JIP-poly, 43.8% had severe synovial lining hyperplasia compared to 14.3% in JIP-oligo (*P* = 0.002). Moreover, 21.9% had severe inflammatory cell infiltration compared to 6.1% in JIP-oligo (*P* = 0.001) and 18.8% had severe stromal density versus 8.2% for JIP-oligo (*P* = 0.034). JIP-hand exhibited increased synovitis levels compared to JIP-oligo for lining layer hyperplasia (27.9% vs. 14.3%; *P* = 0.015) and inflammatory infiltrate (17.4% vs. 6.1%; *P* = 0.013). Furthermore, in JIP-hand, 29.1% had severe stromal density compared to 7.4% in JIP-foot (*P* = 0.211) and 8.2% in JIP-oligo (*P* = 0.013). Although JIP-foot had a similar overall synovitis score as JIP-hand, it was not characterized by any particular KSS component; rather, it had moderate inflammatory activity across all elements.

Since KSS was previously shown to be linked to disease activity^3^, we adjusted for DAS categories to ensure the cluster differences were not merely an effect of the clinical metric. After correction, differences in lining layer hyperplasia (*P* = 0.035) and inflammatory infiltrate (*P* = 0.005) persisted between clusters, while stromal density differences became non-significant (*P* = 0.082).

For a complete overview, we also examined whether sampling from joint areas specific to each JIP phenotype would yield different results compared to using only knee biopsies. We collected samples from lower extremities for JIP-foot patients, knees for JIP-oligo patients, fingers or wrists for JIP-hand patients, and knees or wrists for JIP-poly patients. This targeted sampling approach produced results similar to those found when analyzing knee biopsies alone (Supplementary Fig. 18).

## Discussion

Through deep learning and clustering analysis of real-world clinical data, we identified four distinct RA phenotypes at baseline: foot-dominant (JIP-foot), seropositive oligoarticular (JIP-oligo), seronegative hand-dominant (JIP-hand), and polyarticular (JIP-poly) disease. While our hypothesis-free approach enabled detection of novel non-linear clinical signatures, we mitigated the risk of spurious correlations through rigorous validation, including stability testing, clinical outcome validation, independent cohort replication, and synovial histological correlation.

These results show promising directions for advancing RA management. While not yet clinically applicable, the identified clusters show clinical value as prognostic markers, with hand-foot differentiation proving particularly valuable for predicting clinical outcomes. Both foot-dominant clusters (JIP-foot, JIP-poly) showed higher MTX failure and lower remission rates compared to JIP-hand, independent of baseline joint involvement, symptom duration, or treatment timing. Notably, the impact of foot involvement on treatment failure rivaled that of ACPA-positivity as an independent risk factor, indicating that a more comprehensive joint assessment should be considered in clinical practice.

While this research underscores the importance of foot joints, current joint-specific treatment research often relies on 28-joint counts^27^, overlooking the feet despite their common involvement in RA^28–30^. Notably, our findings validate previous cross-sectional observations of poor prognosis in feet/ankle-involved disease^28^ and support Ciurea et al.‘s observation that foot involvement appears more persistent compared to other anatomical regions^31^. However, this study uniquely demonstrates this association in treatment-naïve patients at clinical presentation.

Nevertheless, dedicated trials are needed to determine whether tailoring therapy to specific patient groups—such as those with foot involvement—can improve outcomes compared to standard care. Recent studies demonstrate that synovial fibroblasts exhibit distinct transcriptomic and epigenomic profiles based on anatomical location, potentially explaining the biological underpinnings responsible for these joint-specific treatment outcomes^32–34^.

Contrary to common assumptions, we did not observe a clear ACPA dichotomy. This finding aligns with previous baseline studies that demonstrate that, despite known differences in risk factors and prognosis between ACPA-positive and ACPA-negative patients, the clinical phenotype at initial diagnosis is similar for both groups^25,35,36^. ACPA prevalence was lowest in typical RA clusters (JIP-hand, JIP-poly) and highest in the oligoarticular cluster (JIP-oligo). While this pattern might partially reflect classification criteria^37^, our use of 1 year diagnosis validation and physician diagnosis in sets A and C minimizes misclassification bias. The high ACPA positivity in JIP-foot corroborates recent findings of increased foot involvement in ACPA-positive patients^38^.

Although seronegative patients have traditionally been considered to have a milder disease course^39^, our study shows this is not the case for the JIP-poly cluster, which is associated with poorer outcomes. In contrast, the seronegative JIP-hand cluster aligns with the expected pattern, showing a milder disease course. Recent literature also challenges the historical assumption of a uniformly mild prognosis in seronegative patients. For example, Duong et al. identified ACPA-positivity as a predictor of better treatment response^40^, and the ARCTIC trial by Haavardsholm et al. found that ACPA-negative patients exhibited slower treatment responses^41^. While our findings suggest that ACPA status may still play a role in disease progression, the relationship appears to be more nuanced and warrants further investigation.

The treatment response disparity between hand and foot clusters was most pronounced in ACPA-positive patients. Across replication sets, we consistently observed significant differences between JIP-hand and JIP-poly, with two of three datasets also showing increased response in JIP-hand versus JIP-foot. For remission, we found that the different cluster-associated outcomes vanished in ACPA-positive patients, possibly reflecting the impact of targeted therapy intensification protocols particularly for ACPA positive patients.

Patients with hand-dominant joint inflammation (JIP-hand) showed surprisingly good outcomes, prompting us to explore several possible explanations. We found that these positive results could not be explained solely by how long patients had symptoms, their disease activity at baseline, or their lower prevalence of ACPA in that cluster. Additionally, we found no increased parvovirus positivity in the JIP-hand group compared to other clusters, indicating that misdiagnosis of RA due to self-limiting reactive arthritis was unlikely^42,43^. One possible explanation is that these patients represent a phenotype of seronegative RA characterized by particular hand and wrist involvement, as reported by Burns et al.^44^. Alternatively, hand-dominant presentations may appear more responsive due to the widespread use of simplified disease indexes in trials (DAS28, CDAI) excluding foot and ankle joints^28,30^, potentially limiting the generalizability to atypical presentations.

Our clusters captured previously described age-related subsets, including elderly-onset RA (EORA) in JIP-hand, characterized by higher inflammation markers, lower female prevalence, and reduced autoantibody positivity^10,11^. However, our analysis revealed more granular subtypes beyond the EORA/YORA dichotomy.

Further analysis of synovial tissue samples revealed distinct histological differences. Both the JIP-poly and JIP-hand groups exhibited severe synovitis. Specifically, JIP-poly was characterized by increased lining hyperplasia and sublining leukocytic infiltration, whereas JIP-hand showed greater stromal density. In contrast, the JIP-oligo group predominantly displayed mild inflammation. These histopathological differences were statistically significant. Notably, after adjusting for disease activity levels, the difference in stromal density was no longer significant, while the differences in inflammatory infiltrate and lining hyperplasia remained significant.

The striking histological differences between our patient clusters further supports the notion that these may represent distinct disease subtypes. The aggressive inflammatory patterns observed in JIP-poly and JIP-hand groups contrast sharply with the mild inflammation seen in JIP-oligo, suggesting different pathological mechanisms.

However, to fully understand the biological basis of these differences, research must extend beyond histopathological features alone. Molecular profiling of synovial tissue offers the potential for deeper mechanistic insights^14^. This approach has proven successful in previous work by Rivellese et al.^45^ who identified distinct molecular pathotypes that predicted differential responses to tocilizumab and rituximab.

A limitation of our study is that we defined MTX success based on changes in medication, which could include switches due to side effects, though this likely underestimates rather than overestimates the observed associations. Center-specific therapeutic approaches varied but did not affect cluster-outcome associations. While temporal cluster stability was not directly assessed, previous evidence of consistent joint involvement patterns and cross-sectional associations support stability over time^46^.

Due to the real-world nature of the data, we could not account for all possible factors of influence. Most notably, missing BMI data may have limited insights into disease heterogeneity. Overweight RA patients show higher disease activity scores and worse treatment outcomes but less radiographic damage^47,48^, suggesting adiposity-related inflammatory pathways and cytokine profiles^47–50^. However, obesity may also inflate subjective clinical measures independent of actual inflammatory activity^48,51^, warranting future investigation.

Another limitation is that we solely focused on knee biopsies due to insufficient data for other joint locations. This prevented us from exploring and comparing different tissue environments even though they might be crucial for understanding the different phenotypes. Additionally, knee biopsies may reflect synovial features from concurrent non-inflammatory conditions such as osteoarthritis, which commonly affects this joint. However, a previous study by Alivernini et al.^3^ demonstrates that Krenn synovitis scores are significantly lower in osteoarthritis biopsies (1.70 ± 0.15), suggesting minimal confounding impact on our assessment of inflammation.

Important to underline is that our identified clusters are not set in stone. Though we observed a high robustness of our clusters, patients lie on a gradient (Supplementary Fig. 4) and did not segregate in clearly separable modules. The cluster structure that we identified could also be summarized into more or fewer clusters and the clusters might become clearer when more layers of information are added. Such types of information could be genetics, gene expression patterns and molecular profiles from blood^14,52^. Despite these limitations, our study demonstrates the value of unsupervised, data-driven approaches in uncovering hidden disease patterns, with joint involvement patterns emerging as a major axis of variation.

In conclusion, our clustering analysis identified four baseline RA phenotypes, each defined by distinct patterns of hand and foot involvement that predict 1 year clinical outcomes and correspond to histological differences. This data-driven approach offers greater granularity than conventional age- or ACPA-based stratifications, suggesting the existence of distinct underlying etiologies that merit further biological investigation.

## Methods

### Patients

Our study comprises three different phases: a (i) developmental phase where we identify and validate subtypes in a discovery set according to long term outcomes (set A), a (ii) replication phase where we cluster novel patients using historic trial- (set B) and external hospital data (set C) to infer generalizability by replicating the treatment analysis and finally (iii) a downstream analysis in external hospital data (set D) where we explore differences between clusters in their synovial tissue.

Set A consisted of 1387 RA-patients that visited the rheumatology outpatient clinic of the Leiden University Medical Centre for the first time between August 29th, 2011 till December 1st 2022. RA diagnosis was based on the physician’s diagnosis within 1 year since first visit^53,54^.

Set B concerned 307 RA-patients from the IMPROVED trial that were recruited between March 2007 till September 2010^55^. This trial recruited undifferentiated arthritis and early RA with <2 years of symptoms. We selected only those patients who met the ACR2010 criteria within 1 year after inclusion. All patients received MTX at baseline and were randomized into two arms of treatment intensification if they did not reach remission after 4 months.

Set C included 515 RA patients from Reumazorg Zuid West Nederland (RZWN), collected between January 2015 and December 1, 2022. These patients were from nine different hospitals across the south west of the Netherlands, with the largest groups coming from Goes (*n* = 157), Roosendaal (*n* = 153), and Vlissingen (*n* = 49). Herein, the diagnosis of RA was defined as having an ICD-code for RA and starting with a conventional DMARD.

Set D included 262 RA patients who met the ACR 2010 criteria for RA, drawn from the SYNGem Biopsy Unit cohort at the Fondazione Policlinico Universitario A. Gemelli IRCCS–Università Cattolica del Sacro Cuore in Rome, Italy. All patients underwent minimally invasive, ultrasound-guided synovial tissue biopsy during their initial rheumatological evaluation as part of routine clinical management^3^. Each tissue sample was processed with Hematoxylin and Eosin staining, and synovitis was graded using the total Krenn Synovitis Score (KSS)^26^.

Across all sets, a minimum follow-up of 1 year was required to ascertain the diagnosis of RA. Prior to conducting the study, we received ethical approval from the Medical Ethics Committee (METC) at Leiden University Medical Center according to study protocol B18.057. The study complied with the Declaration of Helsinki and national guidelines, including the COREON Code of Conduct for Health Research. Patients and the public were not involved in study development, execution, or dissemination.

### Preprocessing of electronic health records

To construct patient phenotypic profiles, we extracted information on serology (RF and ACPA), location of joint involvement (tender- and swollen joints (TJC and SJC)), demographics, blood profiles (hemoglobin, hematocrit, leukocyte- and thrombocytes levels) and ESR at baseline (Supplementary Table 6). Baseline was defined as the first visit to the clinic (set A & C) or the moment of inclusion in the trial (set B). Patients with missing lab or joint location variables were dropped (Supplementary Fig. 1).

We normalized the numerical data using a Yeo-Johnson transformation, except for the ESR levels where we applied a log transformation due to their log-normal distribution. For the categorical data, we implemented one-hot encoding, which created separate binary fields (yes/no) for each possible category value.

### Construction of patient embedding

We integrated the different EHR data types to create a condensed patient representation (called a patient embedding) using a multi-modal autoencoder (MMAE). This MMAE had a narrowing structure with encoder layers containing 128, 64, and finally 8 neurons. For categorical data, we used sigmoid activation functions with Bernoulli loss, while numerical data utilized ReLU activation with Gaussian loss. To prevent overfitting, we compared performance between our training set (80% of data) and validation set (20%), using the Adam optimizer throughout.

After creating the patient embedding, we grouped patients into subcommunities based on clinical similarities using PhenoGraph^56^. We selected PhenoGraph rather than more common methods like K-means clustering because it performs better with our sparse, high-dimensional medical data.

### Cluster interpretation

For each cluster, we examined the characteristics and visualized the phenotype on a pictorial mannequin with an integrated heatmap to demonstrate the number of involved joints. We used a surrogate ML-technique to model the cluster assignment and subjected this model to a SHAP (SHapley Additive exPlanations)^57^ analysis to retrospectively identify the most important variables per cluster. The SHAP plots show the strength and direction of impact of that variable for each patient (also those who are not assigned to that cluster).

### Cluster validation

To confirm that our identified clusters comprised a stable and relevant partitioning, we performed a number of validation checks. We examined cluster stability by measuring how often patients co-cluster across 1000 random subsets of the data and assessed possible factors influencing the partitioning. Next, we conducted a Local Inverse Simpson’s Index analysis^58^ to assess whether physicians are evenly distributed across the clusters or if the patient subgroups merely reflect the reporting differences between physicians (see supplemental material).

To infer the clinical relevance, clinical outcomes were evaluated using a Cox regression model, including: time to MTX-failure (defined by replacement of- or adding an additional DMARD to MTX) and remission (DAS44 < 1.6) within 1 year. Moreover, we evaluated the replicability on an external dataset (set B & set C), where individuals are assigned to clusters in accordance with the previously learned patient embedding (see supplemental material).

### Diversity in synovial characteristics

In addition to the clinical validation, we compared the characteristics of the synovium across the four subgroups, by repeating the clustering analysis in set D (SYNGem cohort). Specifically, we compared the overall Krenn synovitis score (KSS)^26^ and its individual components (lining layer hyperplasia, stromal density and inflammatory infiltrate). Each KSS component was measured on an ordinal scale (0: none, 1: low, 2: moderate, 3: severe). To analyze this, we used ordinal logistic regression, which estimates the odds of transitioning from one severity level to the next, thus relying on sufficient representation. However, since our study focused on treatment-naive RA patients with active synovitis, the lowest level (0: none) for each KSS subitem was rarely observed. As a result, when present, it was combined with the ‘low’ category (1: low).

### Statistical tests

We used the Kruskal–Wallis test followed by Dunn’s post-hoc test to compare numerical values across multiple groups. For survival analysis we used the log-rank test to examine the overall trend and a univariate Cox-regression^59^ to quantify the cluster differences. We inferred the DAS-remission status during the survival analysis, carrying the last observation forward if it was missing (effectively the same as time to event). The proportional hazards assumption was verified by examining the Schoenfeld residuals^60^. We also adjusted the Cox regression model for MTX-response on covariates suspected to influence treatment response (e.g. ACPA positivity, RF positivity, SJC, TJC, Sex and Age). The statistical significance was inferred with ANOVA. For the ordinal values of the KSS components we used an ordered logit, followed up by a post-hoc Wald test to look at individual differences.

### Web Interface

All of our scripts are publicly available online at Github^61^. Moreover, we developed an interactive web tool (https://knevel-lab.github.io/Rheumalyze/) that enables users to map their cohort onto our patient embedding, allowing them to identify the JIP phenotypes present in their data.

## Supplementary information

Supplementary Information

## Data Availability

The datasets generated and analyzed in this study are not publicly available due to strict privacy laws protecting patient data. However, the datasets may be made available by the corresponding author upon reasonable request.

## Code availability

We built an interactive webtool (https://knevel-lab.github.io/Rheumalyze/) that users can use to project their patients on the Leiden data, to infer the JIP-phenotype within their own dataset. We have made our scripts available in a public repository at GitHub^61^.
