# Supplementary material for: Location and amount of joint involvement differentiates rheumatoid arthritis into different clinical subsets
Source: NPJ Digit Med. 2025 Oct 23;8:623. doi: 10.1038/s41746-025-01997-1 (PMC12550013; doi:10.1038/s41746-025-01997-1)
Supplement: Supplementary file 1 — Supplementary Information [file 41746_2025_1997_MOESM1_ESM.pdf]

Supplementary Information File I: Tables

Supplementary material of "**Location and amount of joint involvement differentiates rheumatoid arthritis into different clinical subsets.**" By *Tjardo D. Maarseveen, Marc P. Maurits, Lavinia Agra Coletto, Simone Perniola, Stefan Böhringer, Nils Steinz, Sytske Anne Bergstra, Dario Bruno, Maria Rita Gigante, Viviana A. Pacucci, Luca Petricca, Bianca Boxma-de Klerk, Herman Kasper Glas, Clara Di Mario, Denise Campobasso, Barbara Tolusso, Josien Veris-van Dieren, Annette H. M. van der Helm-van Mil, Elisa Gremese, Maria Antonietta D'Agostino, Marcel J T. Reinders, Marco Gessi, Tom W J Huizinga, Stefano Alivernini, Erik B van den Akker, Rachel Knevel*

Supplementary Table 1: Baseline table for the development set (set A) and the replication sets (set B and C)

	**Set A**	**Set B**	**Set C**
N	1387	307	515
Sex, female* [ɣ] *[N(%)]	894 (64.5)	211 (68.7)	333 (64.7)
Age* [ɣ] *(SD, yr)	60.0 (15.0)	53.1 (14.1)	60.9 (14.1)
RF* [ɣ] *[N(%)]	722 (52.1)	219 (71.3)	322 (62.5)
ACPA* [ɣ] *[N(%)]	676 (48.7)	201 (65.5)	272 (52.8)
ESR* [ɣ] *(IQR, mm/hr)	25 (11-45)	29 (15-42)	16 (8-27)
DAS44(3) (IQR)	3.5 (2.7-4.3)	3.2 (2.8-3.8)	2.8 (1.9-3.9)
SJC (IQR)	7 (4-12)	8 (4-12)	5 (1-13)
TJC (IQR)	10 (5-17)	7 (5-11)	6 (2-14)
DAS28(3) (IQR)	5.2 (4.3-6.2)	5.1 (4.5-5.8)	4.3 (3.4-5.5)
Follow up (IQR, days)	1707 (858-2575)	1388 (1089-1811)	1568 (1012-2174)
Symptom duration (IQR, days)	155 (62-512)	119 (56-214)	n.a.

Where SD= standard deviation; RF= Rheumatoid factor; ACPA= Anti-cyclic citrullinated peptide antibodies; ESR=erythrocyte sedimentation rate; IQR=interquartile range; DAS=Three component disease activity score (either 44 or 28 joint scheme); SJC=swollen joint count; TJC=tender joint count;

Supplementary Table 2: Cluster table for SYNGem cohort used for downstream histopathological analysis.
The clinical variables used for clustering are marked with the gamma symbol (ɣ).

	**Total**	**JIP-Foot**	**JIP-Oligo**	**JIP-Hand**	**JIP-Poly**
N	194	27	49	86	32
Sex, female[ɣ] *[n(%)]*	150 (77.3)	18 (66.7)	41 (83.7)	66 (76.7)	25 (78.1)
Age [ɣ] (SD, yr)	56.1 (14.9)	52.9 (14.0)	51.1 (14.6)	62.1 (12.8)	50.4 (15.1)
KSS	5 (4-7)	5 (4-6)	4 (3-6)	5 (4-7)	6 (5-8)
RF[ɣ] [n(%]	83 (42.8)	8 (29.6)	24 (49.0)	39 (45.3)	12 (37.5)
ACPA[ɣ] [n(%)]	91 (46.9)	11 (40.7)	23 (46.9)	40 (46.5)	17 (53.1)
ESR[ɣ] (IQR, mm/hr)	43 (20-64)	33 (16-52)	32 (14-53)	50 (24-77)	52 (30-66)
SJC (IQR)	10 (7-12)	11 (8-12)	4 (4-6)	10 (8-13)	14 (10-20)
TJC (IQR)	13 (9-16)	14 (12-16)	8 (6-10)	14 (12-16)	19 (14-23)
DAS28 (IQR)	5.5 (4.8-6.7)	5.7 (5.1-6.2)	4.6 (4.1-5.2)	5.8 (5.0-6.8)	6.9 (6.2-7.9)

Where SD= standard deviation; KSS = KRENN synovitis score; RF= Rheumatoid factor; ACPA= Anti-cyclic citrullinated peptide (ACPA) antibodies; ESR=erythrocyte sedimentation rate; IQR=interquartile range; DAS=Three component disease activity score (either 44 or 28 joint scheme); SJC=swollen joint count; TJC=tender joint count; MTX= prevalence of patients receiving methotrexate at baseline;

Supplementary Table 3: Baseline characteristics of the different patient clusters in set A. *The clinical variables used for clustering are marked with the gamma symbol (ɣ).*

	JIP-Foot	JIP-Oligo	JIP-Hand	JIP-Poly	P-value
N	415	380	323	269	
Sex, female ɣ [n(%)]	262 (63.1)	262 (68.9)	198 (61.3)	172 (63.9)	0.165
Age ɣ (SD, yr)	56.8 (14.4)	59.7 (14.9)	68.5 (12.6)	55.1 (14.7)	< 0.001
RF ɣ [n(%)]	245 (59.0)	236 (62.1)	121 (37.5)	120 (44.6)	< 0.001
ACPA ɣ [n(%)]	241 (58.1)	224 (58.9)	97 (30.0)	114 (42.4)	< 0.001
ESR ɣ (IQR, mm/hr)	22 (9-36)	29 (14-46)	31 (17-51)	22 (9-41)	< 0.001
DAS44(3) (IQR)	3.5 (3.0-4.0)	2.5 (2.0-2.8)	3.8 (3.2-4.4)	4.7 (4.0-5.5)	< 0.001
SJC (IQR)	8 (5-11)	3 (1-4)	10 (7-14)	14 (9-21)	< 0.001
TJC (IQR)	11 (8-15)	3 (2-5)	11 (8-15)	24 (18-31)	< 0.001
DAS28(3) (IQR)	5.3 (4.4-6.0)	4.2 (3.4-4.7)	5.6 (5.0-6.4)	6.6 (5.5-7.4)	< 0.001
MTX [n(%)]	354 (85.3)	275 (72.4)	240 (74.3)	218 (81.0)	< 0.001
Follow up (IQR, days)	1749 (860-2663)	1822 (1024-2587)	1267 (706-2179)	2032 (1141-2909)	< 0.001
Symptom duration (IQR, days)	154 (56-365)	217 (63-740)	122 (42-365)	155 (55-365)	0.130

SD= standard deviation; RF= Rheumatoid factor; ACPA= Anti-cyclic citrullinated peptide (ACPA) antibodies; ESR=erythrocyte sedimentation rate; IQR=interquartile range; DAS=Three component disease activity score (either 44 or 28 joint scheme); SJC=swollen joint count; TJC=tender joint count; MTX= prevalence of patients receiving methotrexate at baseline;

Supplementary Table 4: Cluster table for replication set B (IMPROVED trial data). *The clinical variables used for clustering are marked with the gamma symbol (ɣ).*

	JIP-Foot	JIP-Oligo	JIP-Hand	JIP-Poly	P-value
N	90	102	50	65	-
Sex, female $^{\gamma}$ [n(%)]	66 (73.3)	67 (65.7)	28 (56.0)	50 (76.9)	0.068
Age $^{\gamma}$ (SD, yr)	51.0 (13.4)	53.9 (13.5)	58.1 (12.9)	51.3 (15.8)	0.022
RF $^{\gamma}$ [n(%)]	68 (75.6)	75 (73.5)	36 (72.0)	40 (61.5)	0.252
ACPA $^{\gamma}$ [n(%)]	63 (70.0)	71 (69.6)	29 (58.0)	38 (58.5)	0.237
ESR $^{\gamma}$ (IQR, mm/hr)	28 (11-41)	29 (16-40)	32 (16-50)	33 (17-50)	0.488
DAS44(3) (IQR)	3.3 (2.9-3.7)	2.8 (2.5-3.0)	3.3 (3.0-4.0)	4.3 (3.7-4.7)	< 0.001
SJC (IQR)	8 (5-11)	4 (2-6)	9 (6-12)	16 (10-22)	< 0.001
TJC (IQR)	7 (6-10)	5 (3-7)	8 (6-10)	12 (9-15)	< 0.001
DAS28(3) (IQR)	5.0 (4.5-5.8)	4.6 (4.1-5.0)	5.2 (4.8-5.8)	6.1 (5.4-6.7)	< 0.001
Follow up (IQR, days)	2119 (1018-3914)	2553 (1521-4744)	1730 (990-3258)	2177 (1389-4368)	0.915
Symptom duration (IQR, days)	161 (80-281)	135 (67-258)	144 (70-252)	129 (75-252)	0.179

Where SD, standard deviation; RF, rheumatoid factor; ACPA, anti-cyclic citrullinated peptide antibodies; ESR, erythrocyte sedimentation rate; IQR, interquartile range; DAS, three component disease activity score (either 44 or 28 joint scheme); SJC, swollen joint count; TJC, tender joint count;

Supplementary Table 5: Cluster table for replication set C (Reumazorg Zuid West Nederland hospital data). *The clinical variables used for clustering are marked with the gamma symbol (ɣ).*

	**JIP-Foot**	**JIP-Oligo**	**JIP-Hand**	**JIP-Poly**	***P*-value**
N	91	279	77	68	-
Sex, female $^{\gamma}$ [n(%)]	61 (67.0)	176 (63.1)	46 (59.7)	50 (73.5)	0.298
Age $^{\gamma}$ (SD, yr)	61.3 (14.4)	61.2 (14.2)	63.3 (12.9)	56.4 (13.3)	0.103
RF $^{\gamma}$ [n(%)]	57 (62.6)	186 (66.7)	43 (55.8)	36 (52.9)	0.311
ACPA $^{\gamma}$ [n(%)]	51 (56.0)	154 (55.2)	36 (46.8)	31 (45.6)	0.487
ESR $^{\gamma}$ (IQR, mm/hr)	16 (8-26)	17 (8-27)	13 (7-29)	14 (5-30)	0.025
DAS44(3) (IQR)	3.8 (3.2-4.6)	2.2 (1.8-2.6)	3.7 (3.2-4.4)	4.9 (4.2-5.9)	0.737
SJC (IQR)	10 (6-15)	2 (0-4)	14 (10-17)	18 (14-26)	< 0.001
TJC (IQR)	14 (10-18)	3 (1-5)	11 (7-18)	24 (17-38)	< 0.001
DAS28(3) (IQR)	5.3 (4.6-6.3)	3.6 (3.0-4.1)	5.3 (4.7-6.0)	6.3 (5.5-7.3)	< 0.001
Follow up (IQR, days)	1382 (1047-1932)	1515 (968-2183)	1579 (1149-2275)	1843 (1494-2256)	0.029
Symptom duration (IQR, days)	-	-	-	-	-

Supplementary Table 6: All variables per layer in the Electronic Health Records used for clustering set A and the replication sets B and C (= missing for both B&C, **= only missing for set C)*

**LAYER**	**COLUMNS**	
**MANNEQUIN_OHE**	Swollen_elbow_L_neg	Swollen_elbow_L_pos
	Swollen_IP_hand_L_neg	Swollen_IP_hand_L_pos
	Swollen_IP_hand_R_neg	Swollen_IP_hand_R_pos
	Swollen_IP_foot_L_neg	Swollen_IP_foot_L_pos
	Swollen_IP_foot_R_neg	Swollen_IP_foot_R_pos
	Swollen_acromioclaviculair_L_neg	Swollen_acromioclaviculair_L_pos
	Swollen_acromioclaviculair_R_neg	Swollen_acromioclaviculair_R_pos
	Swollen_ankle_L_neg	Swollen_ankle_L_pos
	Swollen_ankle_R_neg	Swollen_ankle_R_pos
	Swollen_cervical spine_neg	Swollen_cervical spine_pos
	Swollen_CMC_L_neg	Swollen_CMC_L_pos
	Swollen_CMC_R_neg	Swollen_CMC_R_pos
	Swollen_DIP_2_hand_L_neg	Swollen_DIP_2_hand_L_pos
	Swollen_DIP_2_foot_L_neg	Swollen_DIP_2_foot_L_pos
	Swollen_DIP_2_hand_R_neg	Swollen_DIP_2_hand_R_pos
	Swollen_DIP_2_foot_R_neg	Swollen_DIP_2_foot_R_pos
	Swollen_DIP_3_hand_L_neg	Swollen_DIP_3_hand_L_pos
	Swollen_DIP_3_foot_L_neg	Swollen_DIP_3_foot_L_pos
	Swollen_DIP_3_hand_R_neg	Swollen_DIP_3_hand_R_pos
	Swollen_DIP_3_foot_R_neg	Swollen_DIP_3_foot_R_pos
	Swollen_DIP_4_hand_L_neg	Swollen_DIP_4_hand_L_pos
	Swollen_DIP_4_foot_L_neg	Swollen_DIP_4_foot_L_pos
	Swollen_DIP_4_hand_R_neg	Swollen_DIP_4_hand_R_pos
	Swollen_DIP_4_foot_R_neg	Swollen_DIP_4_foot_R_pos
	Swollen_DIP_5_hand_L_neg	Swollen_DIP_5_hand_L_pos

	Swollen_DIP_5_foot_L_neg	Swollen_DIP_5_foot_L_pos
	Swollen_DIP_5_hand_R_neg	Swollen_DIP_5_hand_R_pos
	Swollen_DIP_5_foot_R_neg	Swollen_DIP_5_foot_R_pos
	Swollen_elbow_R_neg	Swollen_elbow_R_pos
	Swollen_hip_L_neg	Swollen_hip_L_pos
	Swollen_hip_R_neg	Swollen_hip_R_pos
	Swollen_knee_L_neg	Swollen_knee_L_pos
	Swollen_knee_R_neg	Swollen_knee_R_pos
	Swollen_MCP_1_L_neg	Swollen_MCP_1_L_pos
	Swollen_MCP_1_R_neg	Swollen_MCP_1_R_pos
	Swollen_MCP_2_L_neg	Swollen_MCP_2_L_pos
	Swollen_MCP_2_R_neg	Swollen_MCP_2_R_pos
	Swollen_MCP_3_L_neg	Swollen_MCP_3_L_pos
	Swollen_MCP_3_R_neg	Swollen_MCP_3_R_pos
	Swollen_MCP_4_L_neg	Swollen_MCP_4_L_pos
	Swollen_MCP_4_R_neg	Swollen_MCP_4_R_pos
	Swollen_MCP_5_L_neg	Swollen_MCP_5_L_pos
	Swollen_MCP_5_R_neg	Swollen_MCP_5_R_pos
	Swollen_MTP_1_L_neg	Swollen_MTP_1_L_pos
	Swollen_MTP_1_R_neg	Swollen_MTP_1_R_pos
	Swollen_MTP_2_L_neg	Swollen_MTP_2_L_pos
	Swollen_MTP_2_R_neg	Swollen_MTP_2_R_pos
	Swollen_MTP_3_L_neg	Swollen_MTP_3_L_pos
	Swollen_MTP_3_R_neg	Swollen_MTP_3_R_pos
	Swollen_MTP_4_L_neg	Swollen_MTP_4_L_pos
	Swollen_MTP_4_R_neg	Swollen_MTP_4_R_pos
	Swollen_MTP_5_L_neg	Swollen_MTP_5_L_pos

	Swollen_MTP_5_R_neg	Swollen_MTP_5_R_pos
	Swollen_talo-calcaneo-navicularis_L_neg	Swollen_talo-calcaneo-navicularis_L_pos
	Swollen_talo-calcaneo-navicularis_R_neg	Swollen_talo-calcaneo-navicularis_R_pos
	Swollen_PIP_2_hand_L_neg	Swollen_PIP_2_hand_L_pos
	Swollen_PIP_2_foot_L_neg	Swollen_PIP_2_foot_L_pos
	Swollen_PIP_2_hand_R_neg	Swollen_PIP_2_hand_R_pos
	Swollen_PIP_2_foot_R_neg	Swollen_PIP_2_foot_R_pos
	Swollen_PIP_3_hand_L_neg	Swollen_PIP_3_hand_L_pos
	Swollen_PIP_3_foot_L_neg	Swollen_PIP_3_foot_L_pos
	Swollen_PIP_3_hand_R_neg	Swollen_PIP_3_hand_R_pos
	Swollen_PIP_3_foot_R_neg	Swollen_PIP_3_foot_R_pos
	Swollen_PIP_4_hand_L_neg	Swollen_PIP_4_hand_L_pos
	Swollen_PIP_4_foot_L_neg	Swollen_PIP_4_foot_L_pos
	Swollen_PIP_4_hand_R_neg	Swollen_PIP_4_hand_R_pos
	Swollen_PIP_4_foot_R_neg	Swollen_PIP_4_foot_R_pos
	Swollen_PIP_5_hand_L_neg	Swollen_PIP_5_hand_L_pos
	Swollen_PIP_5_foot_L_neg	Swollen_PIP_5_foot_L_pos
	Swollen_PIP_5_hand_R_neg	Swollen_PIP_5_hand_R_pos
	Swollen_PIP_5_foot_R_neg	Swollen_PIP_5_foot_R_pos
	Swollen_wrist_L_neg	Swollen_wrist_L_pos
	Swollen_wrist_R_neg	Swollen_wrist_R_pos
	Swollen_shoulder_L_neg	Swollen_shoulder_L_pos
	Swollen_shoulder_R_neg	Swollen_shoulder_R_pos
	Swollen_sternoclavicular_L_neg	Swollen_sternoclavicular_L_pos
	Swollen_sternoclavicular_R_neg	Swollen_sternoclavicular_R_pos
	Swollen_tarsometatarsal_L_neg	Swollen_tarsometatarsal_L_pos
	Swollen_tarsometatarsal_R_neg	Swollen_tarsometatarsal_R_pos

	Swollen_temporomandibular_L_neg	Swollen_temporomandibular_L_pos
	Swollen_temporomandibular_R_neg	Swollen_temporomandibular_R_pos
	Tender_elbow_L_neg	Tender_elbow_L_pos
	Tender_IP_hand_L_neg	Tender_IP_hand_L_pos
	Tender_IP_hand_R_neg	Tender_IP_hand_R_pos
	Tender_IP_foot_L_neg	Tender_IP_foot_L_pos
	Tender_IP_foot_R_neg	Tender_IP_foot_R_pos
	Tender_manubriosternal_neg	Tender_manubriosternal_pos
	Tender_acromioclaviculair_L_neg	Tender_acromioclaviculair_L_pos
	Tender_acromioclaviculair_R_neg	Tender_acromioclaviculair_R_pos
	Tender_ankle_L_neg	Tender_ankle_L_pos
	Tender_ankle_R_neg	Tender_ankle_R_pos
	Tender_cervical spine_neg	Tender_cervical spine_pos
	Tender_CMC_L_neg	Tender_CMC_L_pos
	Tender_CMC_R_neg	Tender_CMC_R_pos
	Tender_DIP_2_hand_L_neg	Tender_DIP_2_hand_L_pos
	Tender_DIP_2_foot_L_neg	Tender_DIP_2_foot_L_pos
	Tender_DIP_2_hand_R_neg	Tender_DIP_2_hand_R_pos
	Tender_DIP_2_foot_R_neg	Tender_DIP_2_foot_R_pos
	Tender_DIP_3_hand_L_neg	Tender_DIP_3_hand_L_pos
	Tender_DIP_3_foot_L_neg	Tender_DIP_3_foot_L_pos
	Tender_DIP_3_hand_R_neg	Tender_DIP_3_hand_R_pos
	Tender_DIP_3_foot_R_neg	Tender_DIP_3_foot_R_pos
	Tender_DIP_4_hand_L_neg	Tender_DIP_4_hand_L_pos
	Tender_DIP_4_foot_L_neg	Tender_DIP_4_foot_L_pos
	Tender_DIP_4_hand_R_neg	Tender_DIP_4_hand_R_pos
	Tender_DIP_4_foot_R_neg	Tender_DIP_4_foot_R_pos

	Tender_DIP_5_hand_L_neg	Tender_DIP_5_hand_L_pos
	Tender_DIP_5_foot_L_neg	Tender_DIP_5_foot_L_pos
	Tender_DIP_5_hand_R_neg	Tender_DIP_5_hand_R_pos
	Tender_DIP_5_foot_R_neg	Tender_DIP_5_foot_R_pos
	Tender_elbow_R_neg	Tender_elbow_R_pos
	Tender_hip_L_neg	Tender_hip_L_pos
	Tender_hip_R_neg	Tender_hip_R_pos
	Tender_knee_L_neg	Tender_knee_L_pos
	Tender_knee_R_neg	Tender_knee_R_pos
	Tender_MCP_1_L_neg	Tender_MCP_1_L_pos
	Tender_MCP_1_R_neg	Tender_MCP_1_R_pos
	Tender_MCP_2_L_neg	Tender_MCP_2_L_pos
	Tender_MCP_2_R_neg	Tender_MCP_2_R_pos
	Tender_MCP_3_L_neg	Tender_MCP_3_L_pos
	Tender_MCP_3_R_neg	Tender_MCP_3_R_pos
	Tender_MCP_4_L_neg	Tender_MCP_4_L_pos
	Tender_MCP_4_R_neg	Tender_MCP_4_R_pos
	Tender_MCP_5_L_neg	Tender_MCP_5_L_pos
	Tender_MCP_5_R_neg	Tender_MCP_5_R_pos
	Tender_MTP_1_L_neg	Tender_MTP_1_L_pos
	Tender_MTP_1_R_neg	Tender_MTP_1_R_pos
	Tender_MTP_2_L_neg	Tender_MTP_2_L_pos
	Tender_MTP_2_R_neg	Tender_MTP_2_R_pos
	Tender_MTP_3_L_neg	Tender_MTP_3_L_pos
	Tender_MTP_3_R_neg	Tender_MTP_3_R_pos
	Tender_MTP_4_L_neg	Tender_MTP_4_L_pos
	Tender_MTP_4_R_neg	Tender_MTP_4_R_pos

	Tender_MTP_5_L_neg	Tender_MTP_5_L_pos
	Tender_MTP_5_R_neg	Tender_MTP_5_R_pos
	Tender_talo-calcaneo-navicularis_L_neg	Tender_talo-calcaneo-navicularis_L_pos
	Tender_talo-calcaneo-navicularis_R_neg	Tender_talo-calcaneo-navicularis_R_pos
	Tender_PIP_2_hand_L_neg	Tender_PIP_2_hand_L_pos
	Tender_PIP_2_foot_L_neg	Tender_PIP_2_foot_L_pos
	Tender_PIP_2_hand_R_neg	Tender_PIP_2_hand_R_pos
	Tender_PIP_2_foot_R_neg	Tender_PIP_2_foot_R_pos
	Tender_PIP_3_hand_L_neg	Tender_PIP_3_hand_L_pos
	Tender_PIP_3_foot_L_neg	Tender_PIP_3_foot_L_pos
	Tender_PIP_3_hand_R_neg	Tender_PIP_3_hand_R_pos
	Tender_PIP_3_foot_R_neg	Tender_PIP_3_foot_R_pos
	Tender_PIP_4_hand_L_neg	Tender_PIP_4_hand_L_pos
	Tender_PIP_4_foot_L_neg	Tender_PIP_4_foot_L_pos
	Tender_PIP_4_hand_R_neg	Tender_PIP_4_hand_R_pos
	Tender_PIP_4_foot_R_neg	Tender_PIP_4_foot_R_pos
	Tender_PIP_5_hand_L_neg	Tender_PIP_5_hand_L_pos
	Tender_PIP_5_foot_L_neg	Tender_PIP_5_foot_L_pos
	Tender_PIP_5_hand_R_neg	Tender_PIP_5_hand_R_pos
	Tender_PIP_5_foot_R_neg	Tender_PIP_5_foot_R_pos
	Tender_wrist_L_neg	Tender_wrist_L_pos
	Tender_wrist_R_neg	Tender_wrist_R_pos
	Tender_sacroiliac_L_neg	Tender_sacroiliac_L_pos
	Tender_sacroiliac_R_neg	Tender_sacroiliac_R_pos
	Tender_shoulder_L_neg	Tender_shoulder_L_pos
	Tender_shoulder_R_neg	Tender_shoulder_R_pos
	Tender_sternoclavicular_L_neg	Tender_sternoclavicular_L_pos

	Tender_sternoclavicular_R_neg	Tender_sternoclavicular_R_pos
	Tender_tarsometatarsal_L_neg	Tender_tarsometatarsal_L_pos
	Tender_tarsometatarsal_R_neg	Tender_tarsometatarsal_R_pos
	Tender_temporomandibular_L_neg	Tender_temporomandibular_L_pos
	Tender_temporomandibular_R_neg	Tender_temporomandibular_R_pos
	Swollen_elbow_L_neg	Swollen_elbow_L_pos
	Swollen_IP_hand_L_neg	Swollen_IP_hand_L_pos
	Swollen_IP_hand_R_neg	Swollen_IP_hand_R_pos
	Swollen_IP_foot_L_neg	Swollen_IP_foot_L_pos
	Swollen_IP_foot_R_neg	Swollen_IP_foot_R_pos
	Swollen_acromioclaviculair_L_neg	Swollen_acromioclaviculair_L_pos
	Swollen_acromioclaviculair_R_neg	Swollen_acromioclaviculair_R_pos
	Swollen_ankle_L_neg	Swollen_ankle_L_pos
**SEROLOGY**	ACPA_neg	ACPA_pos
	RF_neg	RF_pos
**LAB**	MCV (Mean Corpuscular Volume)**	MCH (Mean Corpuscular Hemoglobin)*
	MCHC (Mean Corpuscular Hemoglobin Concentration)*	
	Hemoglobin	Hematocrit*
	Leukocytes	Thrombocytes
	ESR (Erytrocyte Sedimentation Rate)	
**DEMOGRAPHICS**	Age	Sex

Where L, left; R, right; neg, negative; pos, positive; IP, interphalangeal; CMC, carpometacarpal; DIP, distal interphalangeal; PIP, proximal Interphalangeal joints; MCP, metacarpophalangeal; MTP, metatarsophalangeal joints; RF, rheumatoid factor; ACPA, anti-cyclic citrullinated peptide (ACPA) antibodies; MCV, mean corpuscular volume; MCH, mean corpuscular hemoglobin; MCHC, mean corpuscular hemoglobin concentration; ESR, erythrocyte sedimentation rate;

Supplementary Information File II: Figures

Supplementary material of **"Location and amount of joint involvement differentiates rheumatoid arthritis into different clinical subsets."** By *Tjardo D. Maarseveen, Marc P. Maurits, Lavinia Agra Coletto, Simone Perniola, Stefan Böhringer, Nils Steinz, Sytske Anne Bergstra, Dario Bruno, Maria Rita Gigante, Viviana A. Pacucci, Luca Petricca, Bianca Boxma-de Klerk, Herman Kasper Glas, Clara Di Mario, Denise Campobasso, Barbara Tolusso, Josien Veris-van Dieren, Annette H. M. van der Helm-van Mil, Elisa Gremese, Maria Antonietta D'Agostino, Marcel J. T. Reinders, Marco Gessi, Tom W J Huizinga, Stefano Alivernini, Erik B van den Akker, Rachel Knevel*

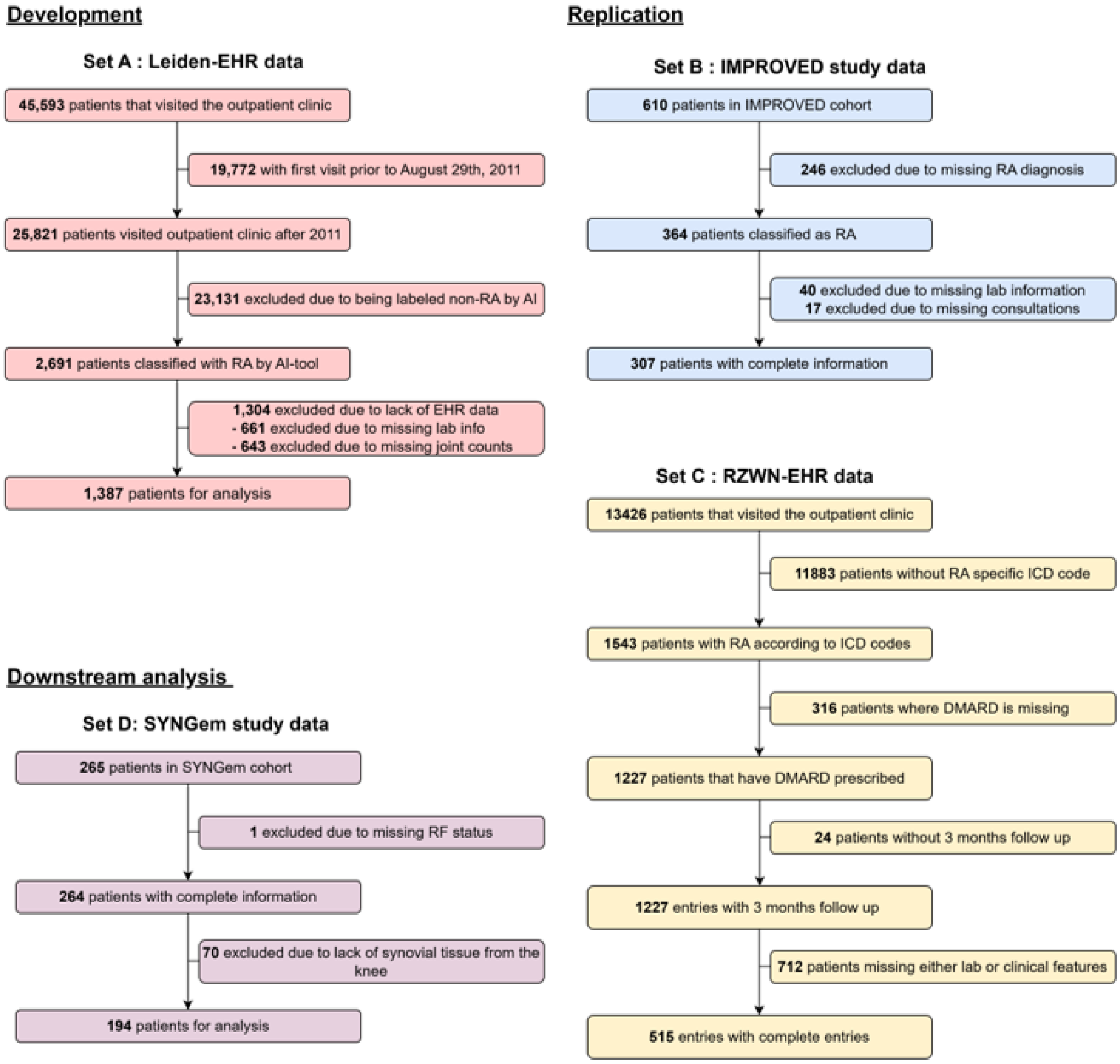

Supplementary Figure 1: Selection of patients for development (set A), replication (set B & C) and downstream analysis into histopathology (set D). *Where Leiden-EHR, Electronic health records repository of Leiden University Medical Center (LUMC); AI, Artificial Intelligence; RZWN-EHR, Electronic health records repository of Reumazorg Zuid West Nederland.*

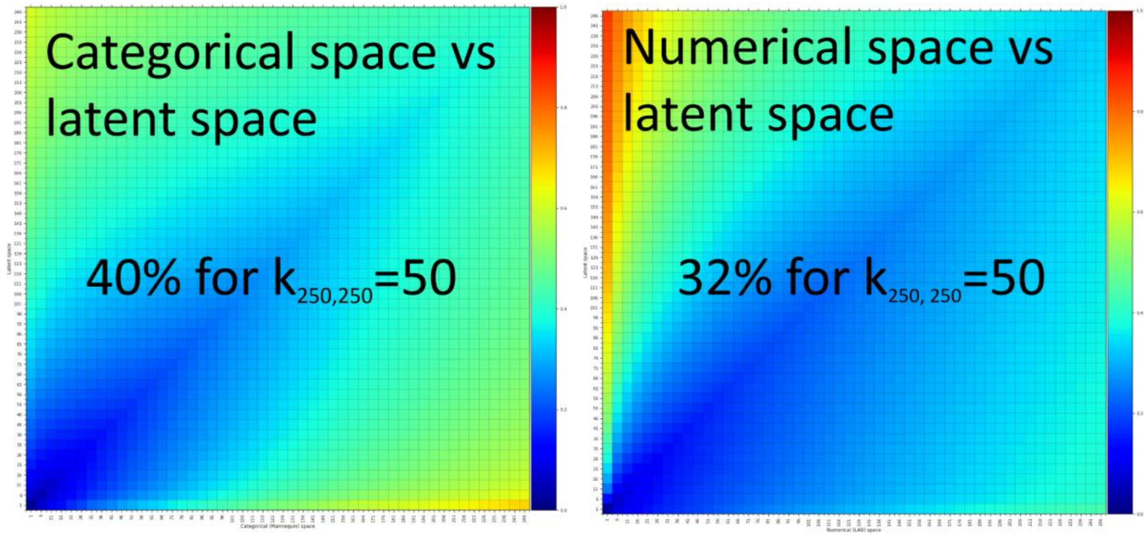

Supplementary Figure 2: Heatmap showing the conservation of local structure from the categorical- and numerical variable sets in the deep learned product space. *The preservation of local structure is measured with the scale dependent similarity measure across different configurations of the k-nearest neighbours algorithm (kNN).*

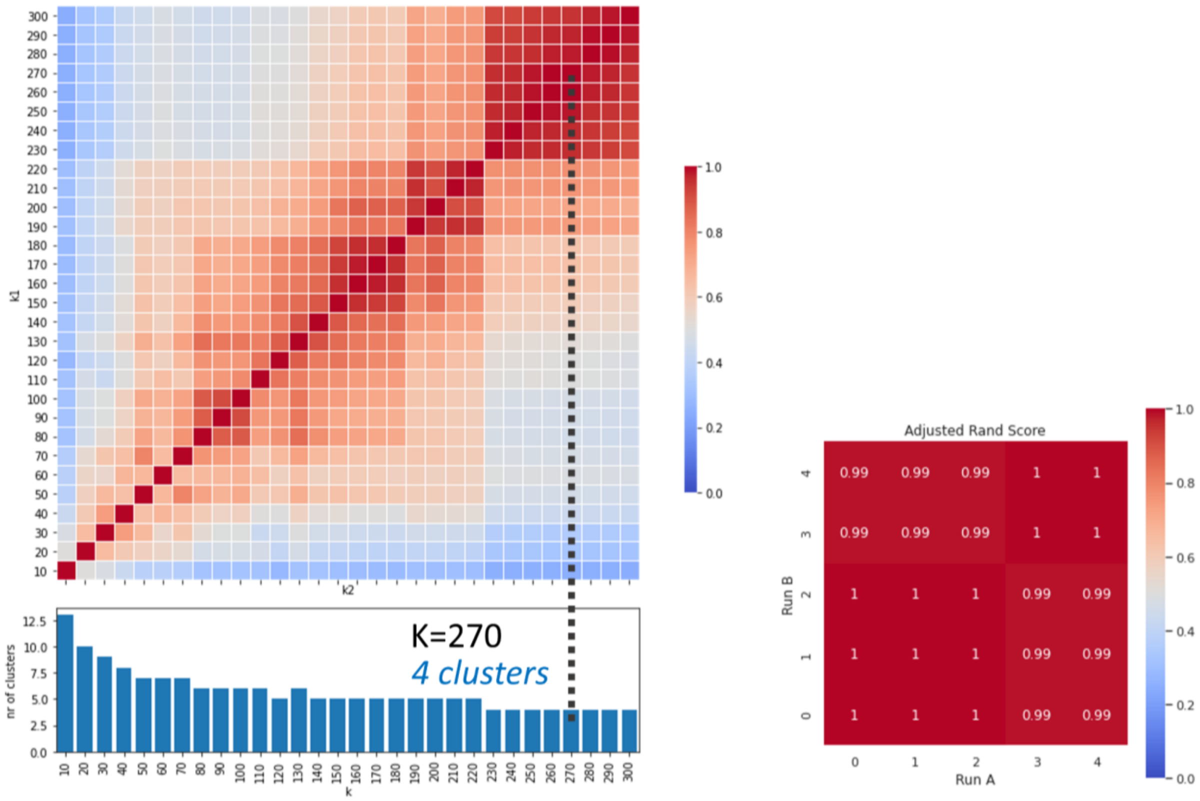

Supplementary Figure 3: Heatmap showing the similarity of clusterings across different configurations of k nearest neighbors (kNN), highlighting the stable and unstable regions for the k-parameter. *The similarity is expressed in adjusted rand index (ARI). The number of clusters associated with each configuration is displayed in the bar chart below. The dashed line indicates our choice of k and the corresponding number of clusters. Whereby k1 = k-value of the first setting of kNN and k2 = k-value of the second setting of kNN.*

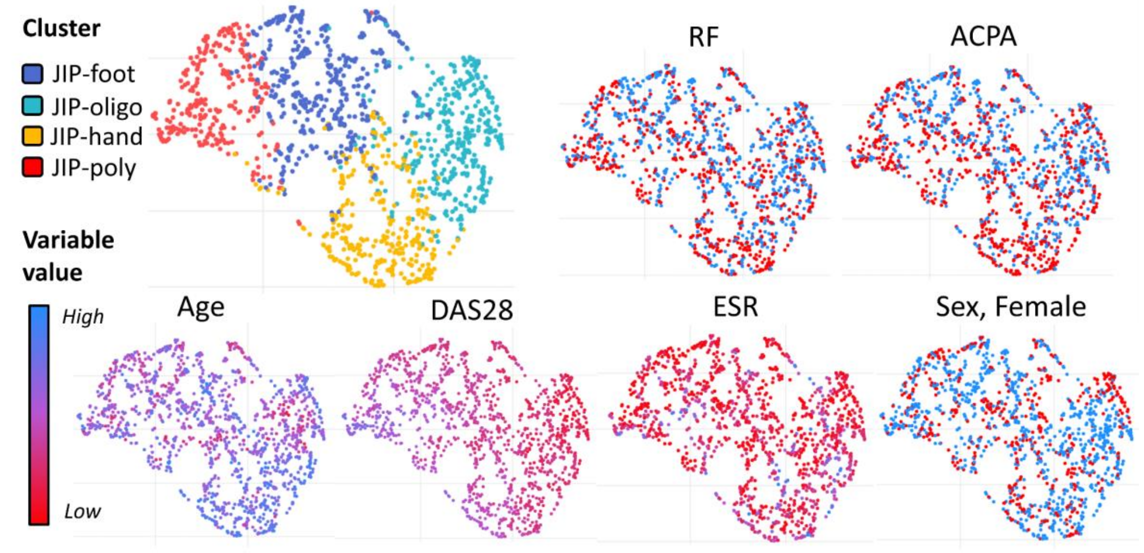

***Supplementary Figure 4: Two-dimensional Uniform Manifold Approximation and Projection (UMAP)-representation of the original patient embedding**. Here each patient is represented by a dot that is colored by the four clusters in the first plot and a gradient from high (blue) to low (red) in the subsequent plots. From left to right: dots are colored on corresponding cluster, Rheumatoid factor (RF) status, anti-cyclic citrullinated protein (ACPA) status, Age, disease activity score 28 (DAS28), erythrocyte sedimentation rate (ESR), Sex (1= Female, 0=Male).*

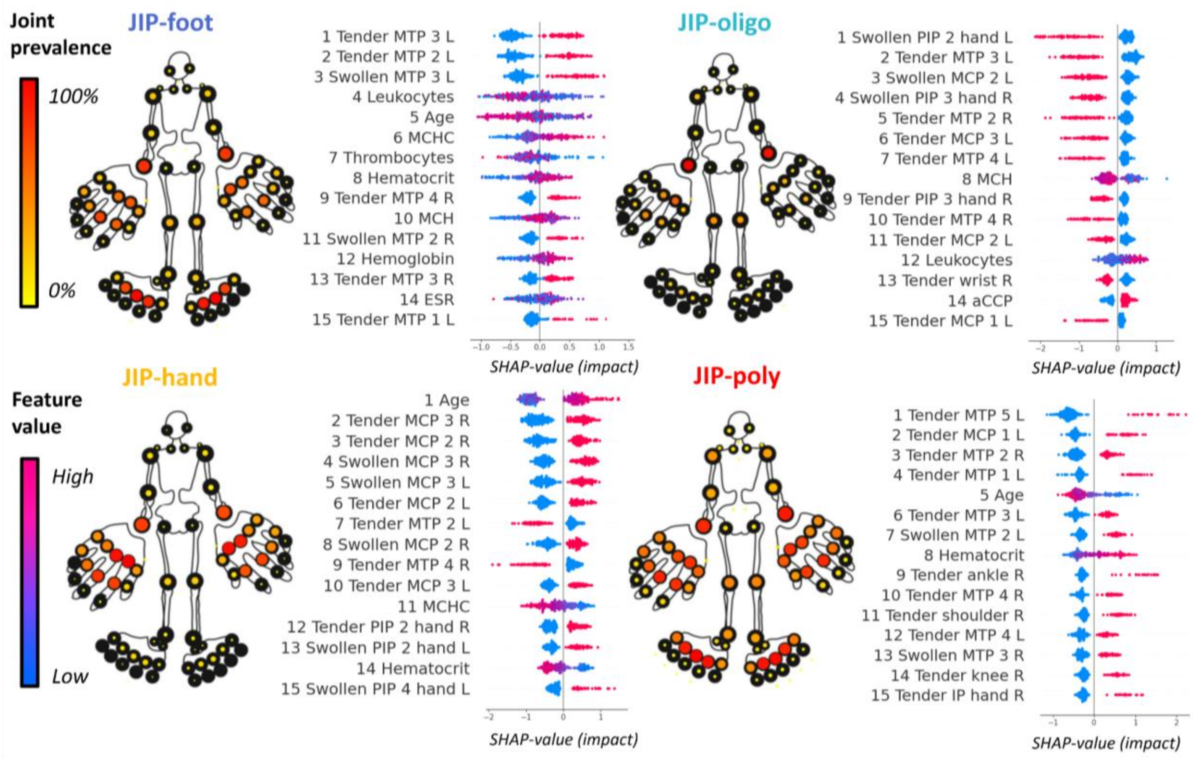

***Supplementary Figure 5: Cluster summary plots showing the average joint involvement, the top 15 driving variables.** The mannequin is formatted as a heatmap, showing the prevalence (red=100%, yellow=0%) of joint involvement (tender or swollen). In the SHAP plots, the most informative features for each cluster are listed in descending order. Here the x-axis shows the strength and direction of impact of that variable for each patient (represented by a dot). The colour of the dot shows the initial value of the clinical variable (pink=high, blue=low). Where ACPA=anti-cyclic citrullinated peptide antibodies; ESR=erythrocyte sedimentation rate; IP= interphalangeal; L=left, MCH=mean corpuscular hemoglobin; MCHC=mean corpuscular hemoglobin*

concentration; MCP=metacarpophalangeal; MTP=metatarsophalangeal; PIP=proximal interphalangeal; R=right;

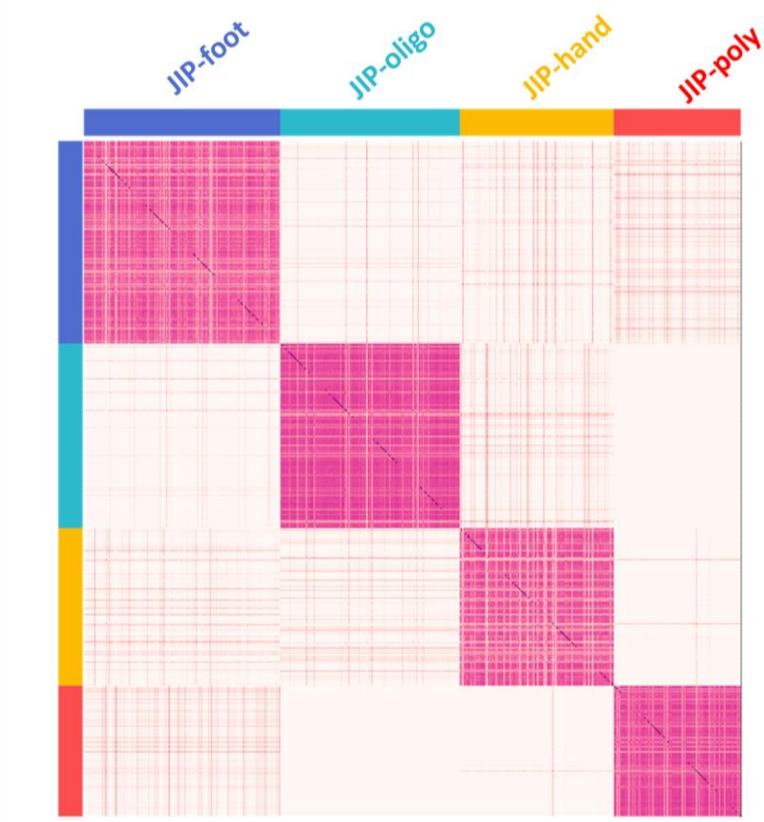

Supplementary Figure 6: Consensus heatmap depicting the cluster stability across 1000 bootstrapped iterations on random subsets of the data. *Pink denotes that patients co-clustered consistently, and white denotes that patients did not co-cluster. Patients are sorted according to their PhenoGraph clusters.*

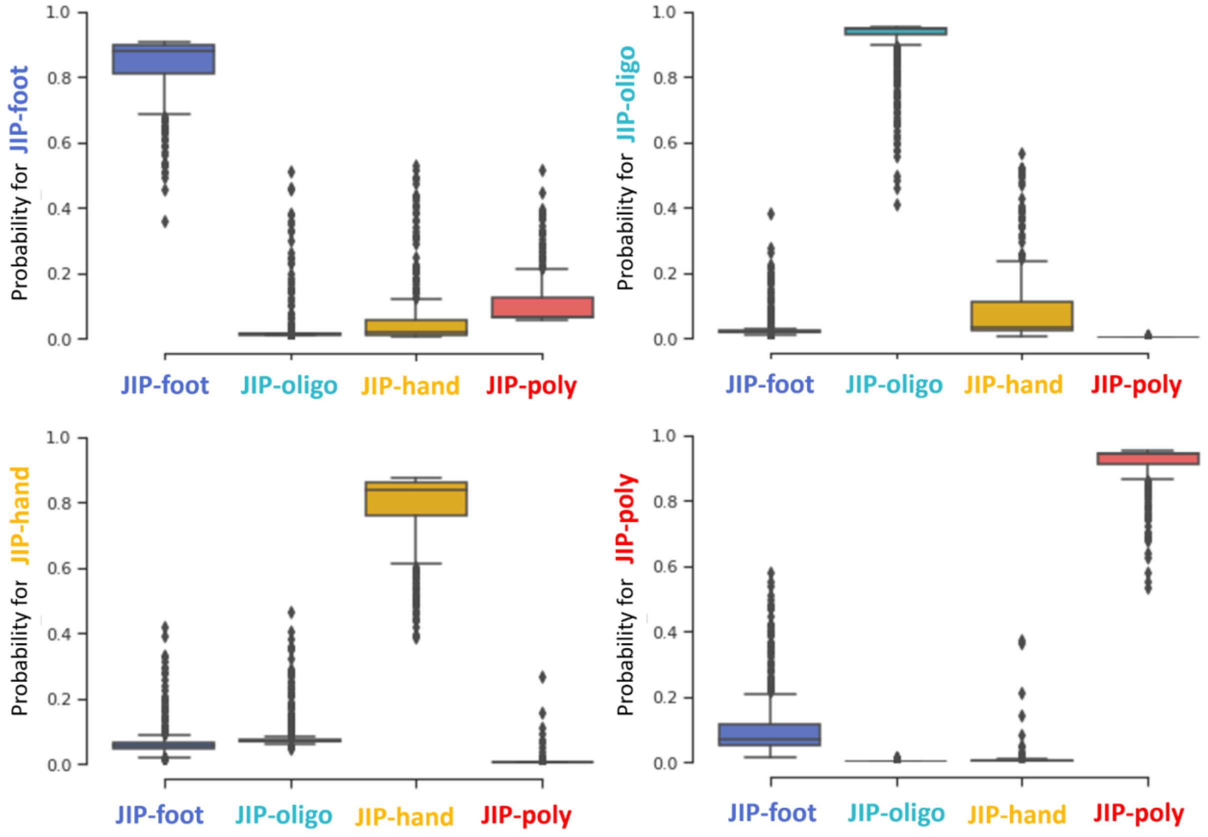

Supplementary Figure 7: Boxplots highlighting probability of patients ending up in the same subset of patients across 1000 bootstrapping iterations.

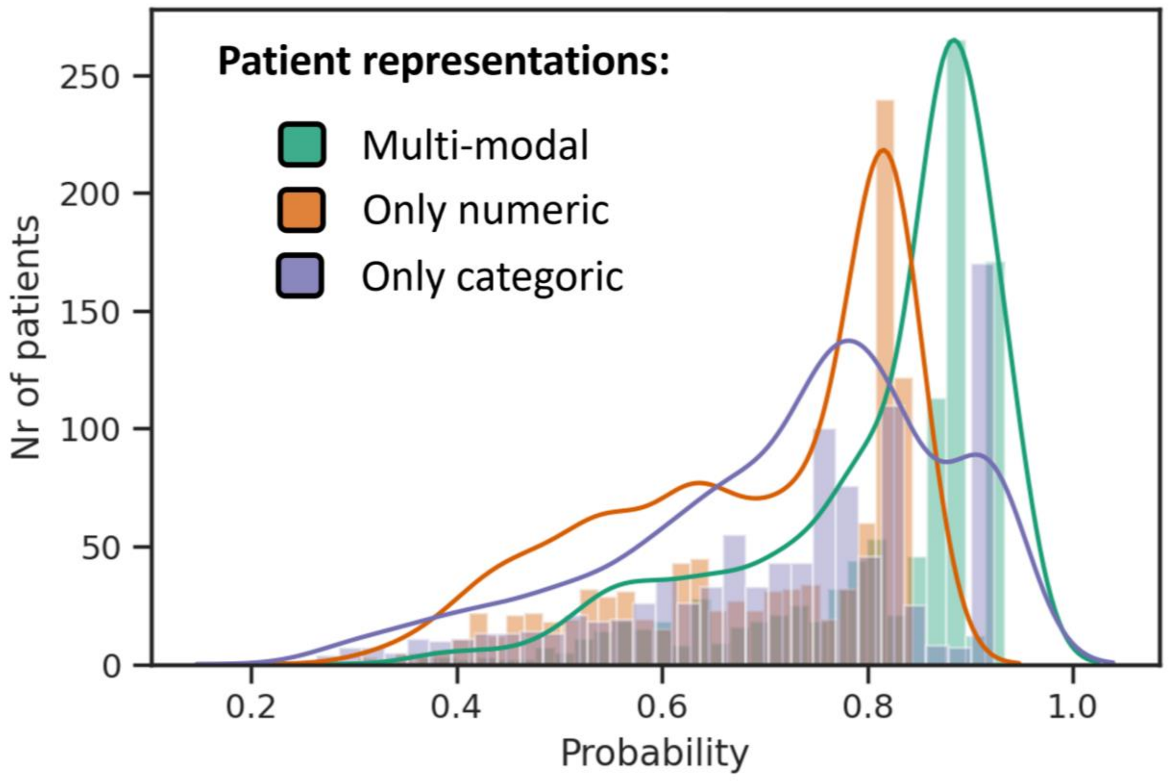

Supplementary Figure 8: Density plot highlighting probability of a patient ending up in the same cluster after bootstrapping 1000 times. *By generating random projections of the data, we can infer the stability of the clusters. We evaluated the stability of clustering for the different data types: numerical (lab, age), categorical (clinical, sex and autoantibodies) and the multi-modal approach that integrates both data types in a single feature space.*

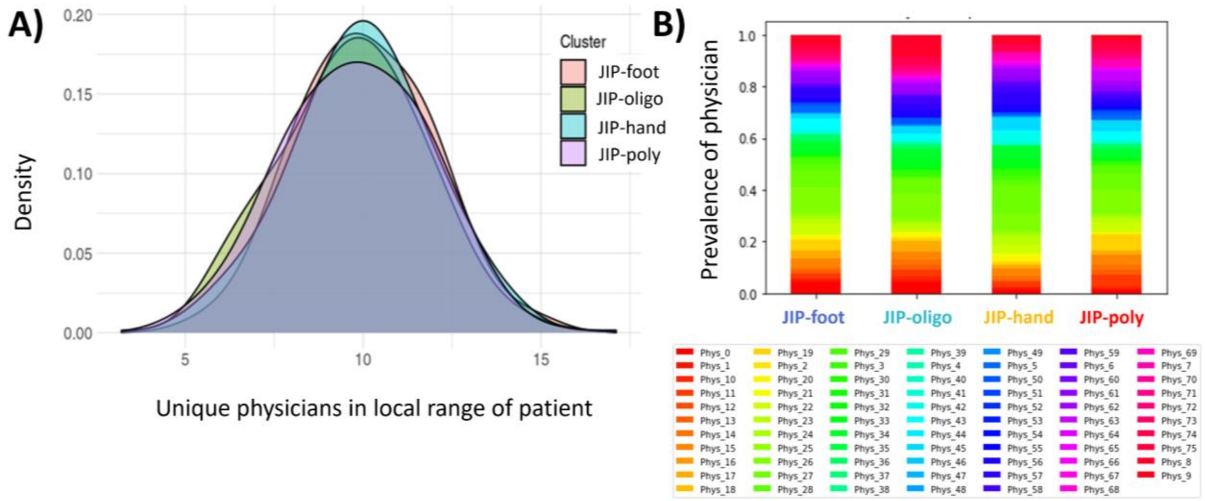

Supplementary Figure 9: Density plot showing the local diversity of physicians per cluster (A) and a stacked bar chart showing the prevalence of physician codes per cluster (B). *The local diversity is quantified with the Local Inverse Simpson's index (LISI), which shows the degree to which the physicians are well-mixed across neighboring data points (n=15 patients). We find that on average patients are surrounded by 10 unique physicians regardless of their cluster.*

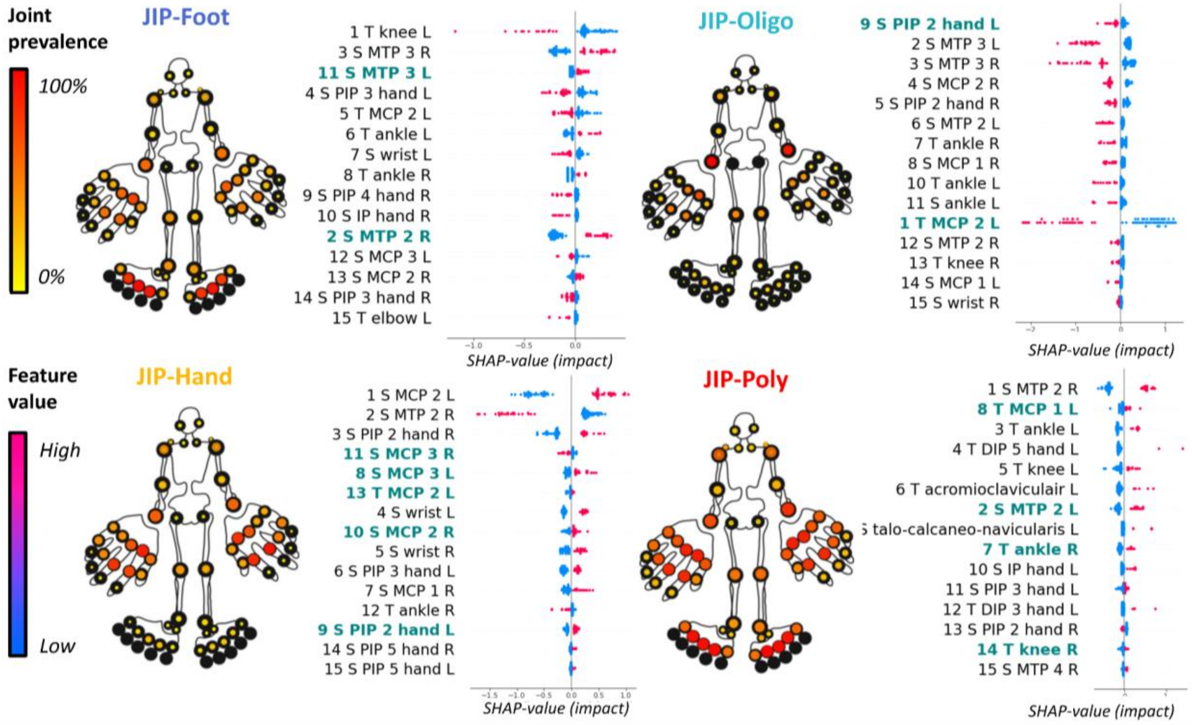

Supplementary Figure 10: Cluster summary plots showing the average joint involvement and the top 15 driving variables in replication set B (IMPROVED). *The mannequin is formatted as a heatmap, showing the prevalence (red=100%, yellow=0%) of joint involvement (tender or swollen). In the SHAP plots, the most informative features for each cluster are listed in descending order. Here the x-axis shows the strength and direction of impact of that variable for each patient (represented by a dot), where the initial value is represented with a color (pink=high, blue=low). The top 15 variables of the replication set are reordered and aligned, if possible, with the original set A (the prefix shows the rank in replication set). Variables that overlap are shown in bold, and colored on agreement (blue= same association, gold= inverse association). Where ACPA, anti-cyclic citrullinated peptide antibodies; ESR, erythrocyte sedimentation rate; IP, interphalangeal; L, left, MCH, mean corpuscular hemoglobin; MCHC, mean corpuscular hemoglobin concentration; MCP, metacarpophalangeal; MTP, metatarsophalangeal; PIP, proximal interphalangeal; R, right;*

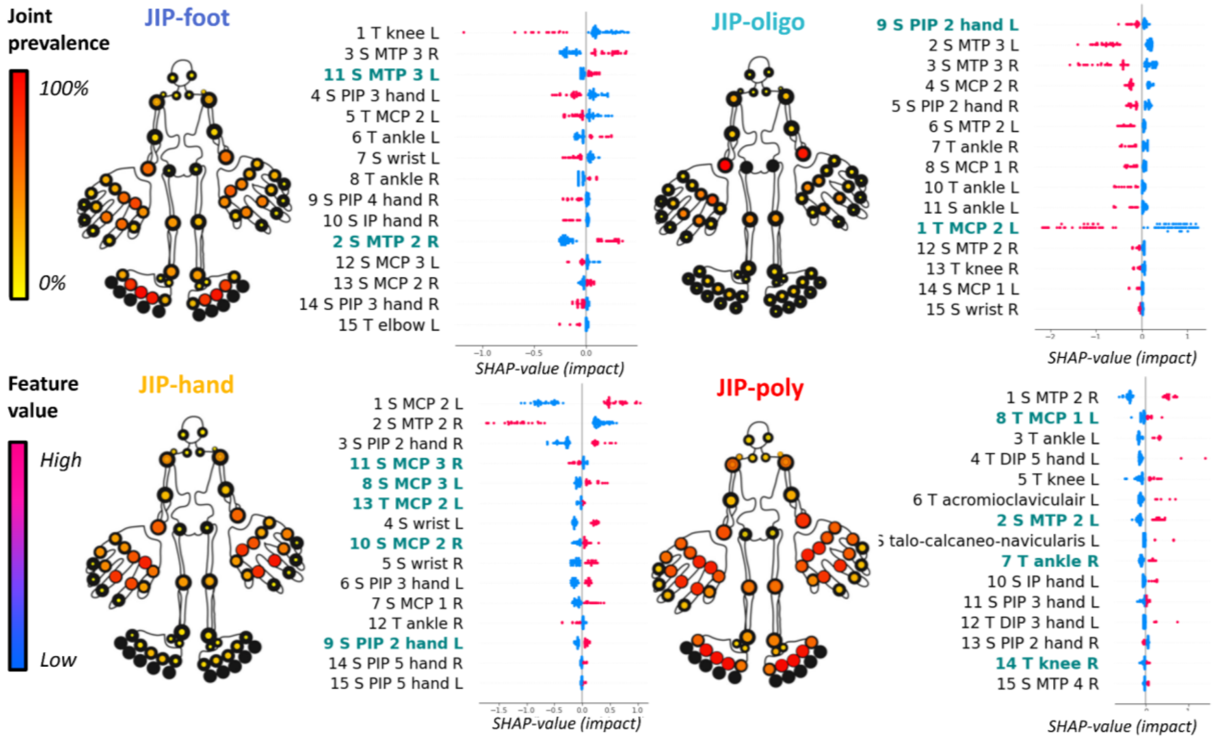

Supplementary Figure 11: Cluster summary plots showing the average joint involvement and the top 15 driving variables in replication set C (RZWN-EHR). *The mannequin is formatted as a heatmap, showing the prevalence (red=100%, yellow=0%) of joint involvement (tender or swollen). In the SHAP plots, the most informative features for each cluster are listed in descending order. Here the x-axis shows the strength and direction of impact of that variable for each patient (represented by a dot), where the initial value is represented with a color (pink=high, blue=low). The top 15 variables of the replication set are reordered and aligned, if possible, with the original set A (the prefix shows the rank in replication set). Variables that overlap are shown in bold, and colored on agreement (blue= same association, gold= inverse association). Where RF, rheumatoid factor; ESR, erythrocyte sedimentation rate; IP, interphalangeal; L, left; MCP, metacarpophalangeal; MTP, metatarsophalangeal; PIP, proximal interphalangeal; R, right;*

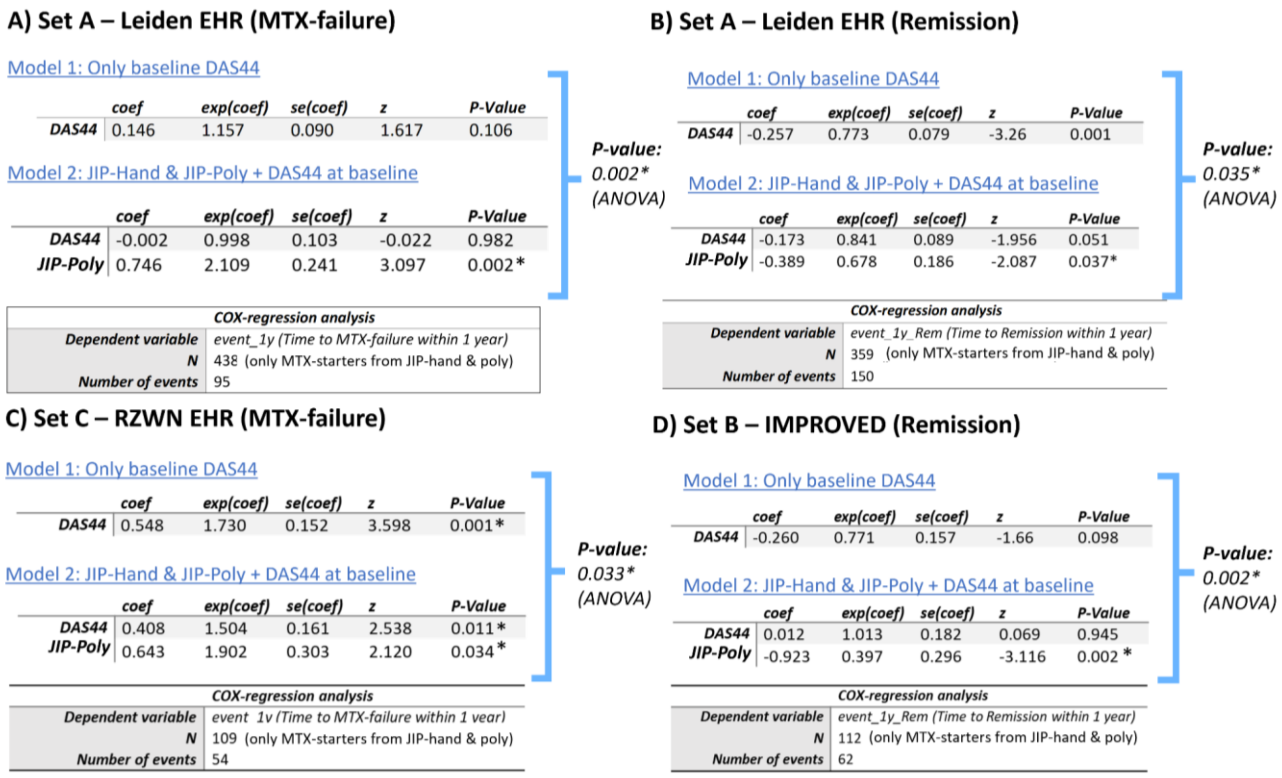

A) Set A – Leiden EHR (MTX-failure)

Model 1: Only baseline DAS44

	coef	exp(coef)	se(coef)	z	P-Value
DAS44	0.146	1.157	0.090	1.617	0.106

Model 2: JIP-Hand & JIP-Poly + DAS44 at baseline

	coef	exp(coef)	se(coef)	z	P-Value
DAS44	-0.002	0.998	0.103	-0.022	0.982
JIP-Poly	0.746	2.109	0.241	3.097	0.002*

P-value: 0.002* (ANOVA)

COX-regression analysis	
Dependent variable	event_1y (Time to MTX-failure within 1 year)
N	438 (only MTX-starters from JIP-hand & poly)
Number of events	95

B) Set A – Leiden EHR (Remission)

Model 1: Only baseline DAS44

	coef	exp(coef)	se(coef)	z	P-Value
DAS44	-0.257	0.773	0.079	-3.26	0.001

Model 2: JIP-Hand & JIP-Poly + DAS44 at baseline

	coef	exp(coef)	se(coef)	z	P-Value
DAS44	-0.173	0.841	0.089	-1.956	0.051
JIP-Poly	-0.389	0.678	0.186	-2.087	0.037*

P-value: 0.035* (ANOVA)

COX-regression analysis	
Dependent variable	event_1y_Rem (Time to Remission within 1 year)
N	359 (only MTX-starters from JIP-hand & poly)
Number of events	150

C) Set C – RZWN EHR (MTX-failure)

Model 1: Only baseline DAS44

	coef	exp(coef)	se(coef)	z	P-Value
DAS44	0.548	1.730	0.152	3.598	0.001*

Model 2: JIP-Hand & JIP-Poly + DAS44 at baseline

	coef	exp(coef)	se(coef)	z	P-Value
DAS44	0.408	1.504	0.161	2.538	0.011*
JIP-Poly	0.643	1.902	0.303	2.120	0.034*

P-value: 0.033* (ANOVA)

COX-regression analysis	
Dependent variable	event_1y (Time to MTX-failure within 1 year)
N	109 (only MTX-starters from JIP-hand & poly)
Number of events	54

D) Set B – IMPROVED (Remission)

Model 1: Only baseline DAS44

	coef	exp(coef)	se(coef)	z	P-Value
DAS44	-0.260	0.771	0.157	-1.66	0.098

Model 2: JIP-Hand & JIP-Poly + DAS44 at baseline

	coef	exp(coef)	se(coef)	z	P-Value
DAS44	0.012	1.013	0.182	0.069	0.945
JIP-Poly	-0.923	0.397	0.296	-3.116	0.002*

P-value: 0.002* (ANOVA)

COX-regression analysis	
Dependent variable	event_1y_Rem (Time to Remission within 1 year)
N	112 (only MTX-starters from JIP-hand & poly)
Number of events	62

Supplementary Figure 12: Comparison of different Cox-regression models to illustrate additive value of clusters on top of baseline DAS44 for predicting difference in time till MTX failure or Remission of JIP-hand vs JIP-poly. *Where model 1 shows the variance explained by baseline DAS44 and model 2 the additive effect of clustering on top of DAS44. Model 1 and model 2 are compared with an ANOVA (Analysis of variance) based on chi-squared test.*

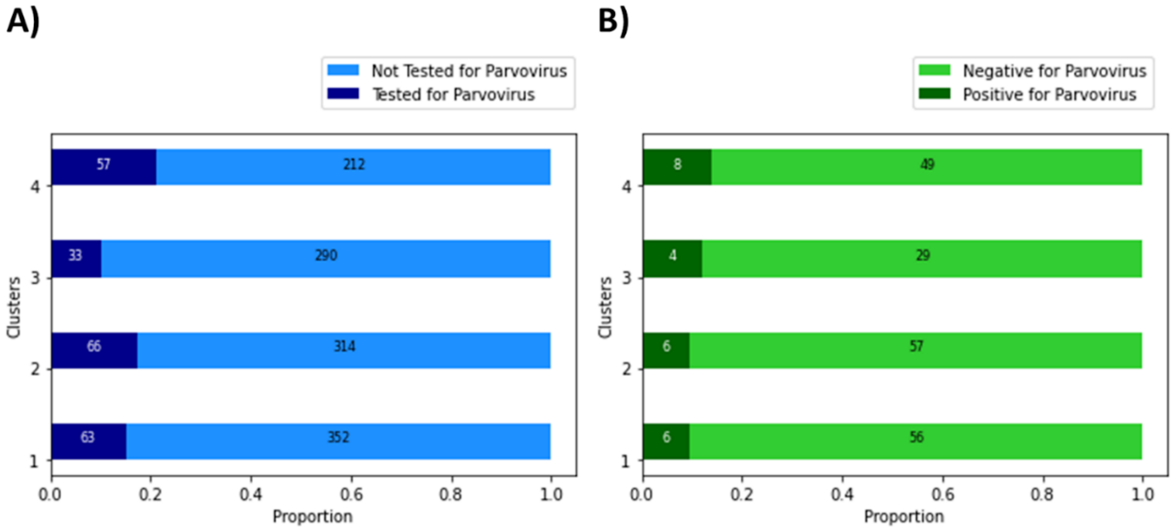

Supplementary Figure 13: Barchart depicting number of patients tested for parvovirus (A), and from those patients the number that tested positive (B) in the Leiden EHR dataset.

JIP-hand vs JIP-poly | JIP-hand vs JIP-foot

A) Set A – Leiden EHR

JIP-hand vs JIP-poly

Model 1: Only baseline DAS44

	coef	exp(coef)	se(coef)	z	P-Value
DAS44	0.265	1.303	0.146	1.812	0.07

Model 2: JIP-Hand & JIP-Poly + DAS44 at baseline

	coef	exp(coef)	se(coef)	Z	P-Value
DAS44	0.089	1.093	0.161	0.550	0.582
JIP-Hand	-1.024	0.359	0.434	-2.350	0.018*

P-value: 0.002* (ANOVA)

COX-regression analysis	
Dependent variable	event_1y (Time to MTX-failure within 1 year)
N	167 (ACPA positive patients from JIP-hand & poly)
Number of events	36

JIP-hand vs JIP-foot

Model 1: Only baseline DAS44

	coef	exp(coef)	se(coef)	z	P-Value
DAS44	-0.034	0.967	0.134	-0.252	0.801

Model 2: JIP-Foot & JIP-Hand + DAS44 at baseline

	coef	exp(coef)	se(coef)	Z	P-Value
DAS44	0.021	1.020	0.138	0.150	0.881
JIP-Hand	-1.162	0.313	0.375	-3.101	0.002*

P-value: <0.001 (ANOVA)

COX-regression analysis	
Dependent variable	event_1y (Time to MTX-failure within 1 year)
N	306 (ACPA positive patients from JIP-foot & hand)
Number of events	78

B) Set B – IMPROVED

JIP-hand vs JIP-poly

Model 1: Only baseline DAS44

	coef (CI 95%)	std error	Z	P-Value
Intercept	-0.991 (-3.506, 1.523)	1.283	-0.773	0.440
DAS44	0.216 (-0.433,0.864)	0.331	0.651	0.515

Model 2: JIP-Hand & JIP-Poly + DAS44 at baseline

	coef (CI 95%)	std error	Z	P-Value
Intercept	1.863 (-1.517, 5.242)	2.186	1.080	0.280
JIP-Hand	-1.834 (-3.214, -0.455)	0.704	-2.606	0.009*
DAS44	-0.345 (-0.448, -1.138)	0.405	-0.853	0.394

P-value: 0.006* (X^2-test)

Logit regression analysis	
Dependent variable	event_1y_bin (MTX-failure within 1 year)
N	64 (ACPA positive patients from JIP-hand & poly)
Number of events	26

JIP-hand vs JIP-foot

Model 1: Only baseline DAS44

	coef (CI 95%)	std error	Z	P-Value
Intercept	-1.425 (-4.167, 1.317)	1.399	-1.019	0.308
DAS44	0.107 (-0.733, 0.947)	0.429	0.250	0.803

Model 2: JIP-Foot & JIP-Hand + DAS44 at baseline

	coef (CI 95%)	std error	Z	P-Value
Intercept	-1.425 (-4.168, 1.318)	1.399	-1.018	0.309
JIP-Hand	-0.039 (-1.141, 1.063)	0.562	-0.069	0.945
DAS44	0.110 (-0.735, 0.956)	0.432	0.256	0.798

P-value: 0.945 (X^2-test)

Logit regression analysis	
Dependent variable	event_1y_bin (MTX-failure within 1 year)
N	88 (ACPA positive patients from JIP-foot & hand)
Number of events	21

C) Set C – RZWN EHR

JIP-hand vs JIP-poly

Model 1: Only baseline DAS44

	coef	exp(coef)	se(coef)	z	P-Value
DAS44	0.603	1.828	0.295	2.044	0.041*

Model 2: JIP-Hand & JIP-Poly + DAS44 at baseline

	coef	exp(coef)	se(coef)	z	P-Value
DAS44	0.559	1.748	0.271	-2.063	0.039 *
JIP-Hand	-1.100	0.333	0.491	-2.240	0.025 *

P-value: 0.019* (ANOVA)

COX-regression analysis	
Dependent variable	event_1y (Time to MTX-failure within 1 year)
N	52 (ACPA positive patients from JIP-hand & poly)
Number of events	20

JIP-hand vs JIP-foot

Model 1: Only baseline DAS44

	coef	exp(coef)	se(coef)	z	P-Value
DAS44	-0.215	0.806	0.222	-0.968	0.333

Model 2: JIP-Foot & JIP-Hand + DAS44 at baseline

	coef	exp(coef)	se(coef)	z	P-Value
DAS44	-0.077	0.926	0.231	-0.335	0.738
JIP-Hand	-0.945	0.389	0.477	-1.980	0.0[illegible]8*

P-value: 0.035* (ANOVA)

COX-regression analysis	
Dependent variable	event_1y (Time to MTX-failure within 1 year)
N	66 (ACPA positive patients from JIP-foot & hand)
Number of events	27

Supplementary Figure 14: Comparison of different regression models to illustrate additive value of clusters on top of baseline DAS44 for predicting MTX failure within ACPA positive stratum in A) Leiden hospital data B) IMPROVED trial data and C) Reumazorg Zuid West Nederland hospital data. *Where we model the MTX-failure according to the difference between JIP-hand and JIP-poly or JIP-foot with a Cox-regression (set A and C) or Logit regression technique (set B) in case of a fixed time of treatment switch. Model 1 shows the variance explained by baseline DAS44 and model 2 the additive effect of clustering on top of DAS44. Model 1 and model 2 are compared with an ANOVA (Analysis of variance) based on chi-squared test.*

JIP-hand vs JIP-poly

A) Set A – Leiden EHR

Model 1: Only symptom duration

	Coef	exp(coef)	se(coef)	z	P-Value
Symptom duration	<0.001	1.000	<0.001	0.937	0.349

Model 2: JIP-Hand & JIP-Poly + Symptom duration

	coef	exp(coef)	se(coef)	z	P-Value
JIP-Hand	0.906	2.474	0.277	3.263	0.001*
Symptom duration	<0.001	1.000	5.115	1.156	0.248

	Cox-regression analysis
Dependent variable	*event_1y (Time to MTX-failure within 1 year)*
N	269 (only those annotated for symptom duration)
Number of events	61

P-value: >0.001* (ANOVA)

JIP-hand vs JIP-foot

Model 1: Only symptom duration

	coef	exp(coef)	se(coef)	z	P-Value
Symptom duration	<0.001	1.000	<0.001	1.280	0.201

Model 2: JIP-Foot & JIP-Hand + Symptom duration

	coef	exp(coef)	se(coef)	z	P-Value
JIP-Hand	-0.788	0.455	0.266	-2.968	0.003*
Symptom duration	<0.001	1.000	<0.001	1.421	0.155

	Cox-regression analysis
Dependent variable	*event_1y (Time to MTX-failure within 1 year)*
N	339 (only those annotated for symptom duration)
Number of events	76

P-value: 0.002* (ANOVA)

B) Set B – IMPROVED

JIP-hand vs JIP-poly

Model 1: Only symptom duration

	Coef (CI 95%)	std error	z	P-Value
Intercept	-0.212 (-0.767, 0.343)	0.283	-0.747	0.455
Symptom duration	0.004 (-0.002, 0.003)	0.001	0.270	0.787

Model 2: JIP-Hand & JIP-Poly + Symptom duration

	Coef (CI 95%)	std error	z	P-Value
Intercept	0.441 (-0.270, 1.152)	0.363	1.215	0.224
JIP-Hand	-1.305 (-2.150, -0.460)	0.431	-3.028	0.002*
Symptom duration	-0.001 (-0.003, 0.002)	0.001	-0.207	0.836

	Logit-regression analysis
Dependent variable	*event_1y_bin (Time to MTX-failure within 1 year)*
N	102 (only MTX-starters)
Number of events	47

P-value: 0.004* (X^2-test)

JIP-hand vs JIP-foot

Model 1: Only symptom duration

	Coef (CI 95%)	std error	z	P-Value
Intercept	-1.321 (-1.926,-0.716)	0.309	-4.281	<0.001*
Symptom duration	0.002 (-0.001, 0.004)	0.001	1.353	0.176

Model 2: JIP-Foot & JIP-Hand + Symptom duration

	Coef (CI 95%)	std error	z	P-Value
Intercept	-1.455 (-2.180, -0.729)	0.370	-3.929	<0.001*
JIP-Hand	0.292 (-0.544, 1.127)	0.426	0.684	0.494
Symptom duration	0.002 (-0.001, 0.005)	0.001	1.449	0.147

	Logit-regression analysis
Dependent variable	*event_1y_bin (Time to MTX-failure within 1 year)*
N	128 (only MTX-starters)
Number of events	34

P-value: 0.819 (X^2-test)

Supplementary Figure 15: Comparison of different Cox-regression models to illustrate additive value of clusters on top of symptom duration for prediction of time till MTX failure. *Where we model the MTX-failure according to the difference between JIP-hand and JIP-poly or JIP-foot with a Cox-regression (set A) or Logit regression technique (set B) in case of a fixed time of treatment switch. Model 1 shows the variance explained by symptom duration and model 2 the additive effect of clustering on top of symptom duration. Model 1 and model 2 are compared with an ANOVA (Analysis of variance) or chi-squared test.*

A) Set A – Leiden EHR: MTX-failure Cox adjusted for prognostic markers

	Coef	Exp(Coef)	Se(Coef)	Z	P-Value
RF	-0.236	0.790	0.171	-1.379	0.168
ACPA	0.347	1.415	0.172	2.020	0.043*
Age	-0.008	0.992	0.005	-1.776	0.076
Sex, F	0.150	1.161	0.140	1.066	0.287
SJC	-0.004	0.996	0.013	-0.339	0.734
TJC	-0.004	0.995	0.011	-0.418	0.676
ESR	0.006	1.006	0.002	2.602	0.009*
JIP-Foot	-0.225	0.798	0.207	-1.087	0.277
JIP-Oligo	-0.529	0.589	0.292	-1.811	0.070
JIP-Hand	-0.750	0.472	0.263	-2.854	0.004*
JIP-Poly	NA	NA	NA	NA	NA

1 / 2 — *P-value: 0.020* (ANOVA)*

COX-regression analysis	
Dependent variable	event_1y (Time to MTX-failure within 1 year)
N	1087 (only MTX-starters)
Number of events	247

B) Set C –RZWN EHR: MTX-failure Cox adjusted for prognostic markers

	Coef	Exp(coef)	Se(coef)	Z	P-Value
RF	-0.699	0.497	0.318	-2.197	0.028*
ACPA	0.269	1.308	0.314	0.856	0.392
Age	-0.018	0.982	0.006	-3.112	0.002*
Sex, F	-0.057	1.059	0.172	0.333	0.739
SJC	-0.003	0.997	0.019	-0.163	0.871
TJC	0.044	1.046	0.017	2.706	-0.007*
ESR	-0.002	0.998	0.004	-0.434	0.664
JIP-Foot	-0.169	0.844	0.272	-0.621	0.535
JIP-Oligo	-0.588	0.555	0.298	-1.974	0.048*
JIP-Hand	-0.735	0.480	0.286	-2.573	0.010*
JIP-Poly	NA	NA	NA	NA	NA

1 / 2 — *P-value: 0.019* (ANOVA)*

COX-regression analysis	
Dependent variable	event_1y (Time to MTX-failure within 1 year)
N	406 (only MTX-starters)
Number of events	161

Supplementary Figure 16: Comparison of Cox regression models for predicting MTX-failure after 1 year based on baseline predictors and cluster information. *To demonstrate the additive effects of our subsets, we compared our full model 2 (including cluster information) to our reduced model 1, which constitutes the known predictors of MTX failure for both the A) original dataset, and the B) replication set. Statistical significance between models was inferred with ANOVA, where p<0.05 is marked with *. Where Coef, regression coefficient; Exp(Coef), exponential of regression coefficient (also known as hazard ratio); RF, rheumatoid factor; ACPA, anti-cyclic citrullinated peptide (ACPA) antibodies; ESR, erythrocyte sedimentation rate; SJC, swollen joint count; TJC, tender joint count; NA, not available;*

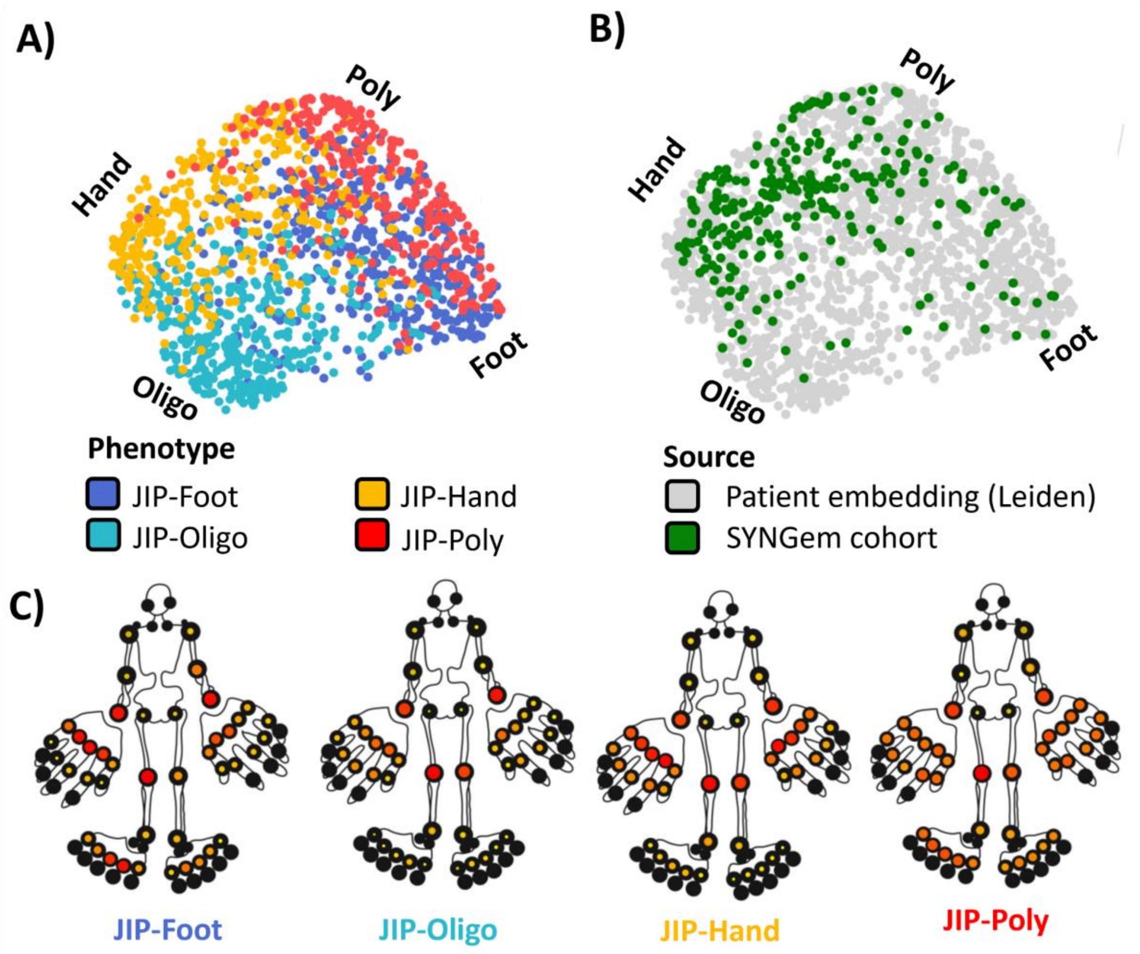

Supplementary Figure 17: Projection of SYNGem cohort patients onto the Leiden patient embedding (set A). *Here, we see* ***a)*** *the original distribution of clusters across the shared product space of Leiden EHR data,* ***b)*** *the orientation of the projected SYNGem cohort patients in darkgreen vs original patients in grey,* ***c)*** *the average joint involvement profiles of the clustered SYNGem patients (C).*

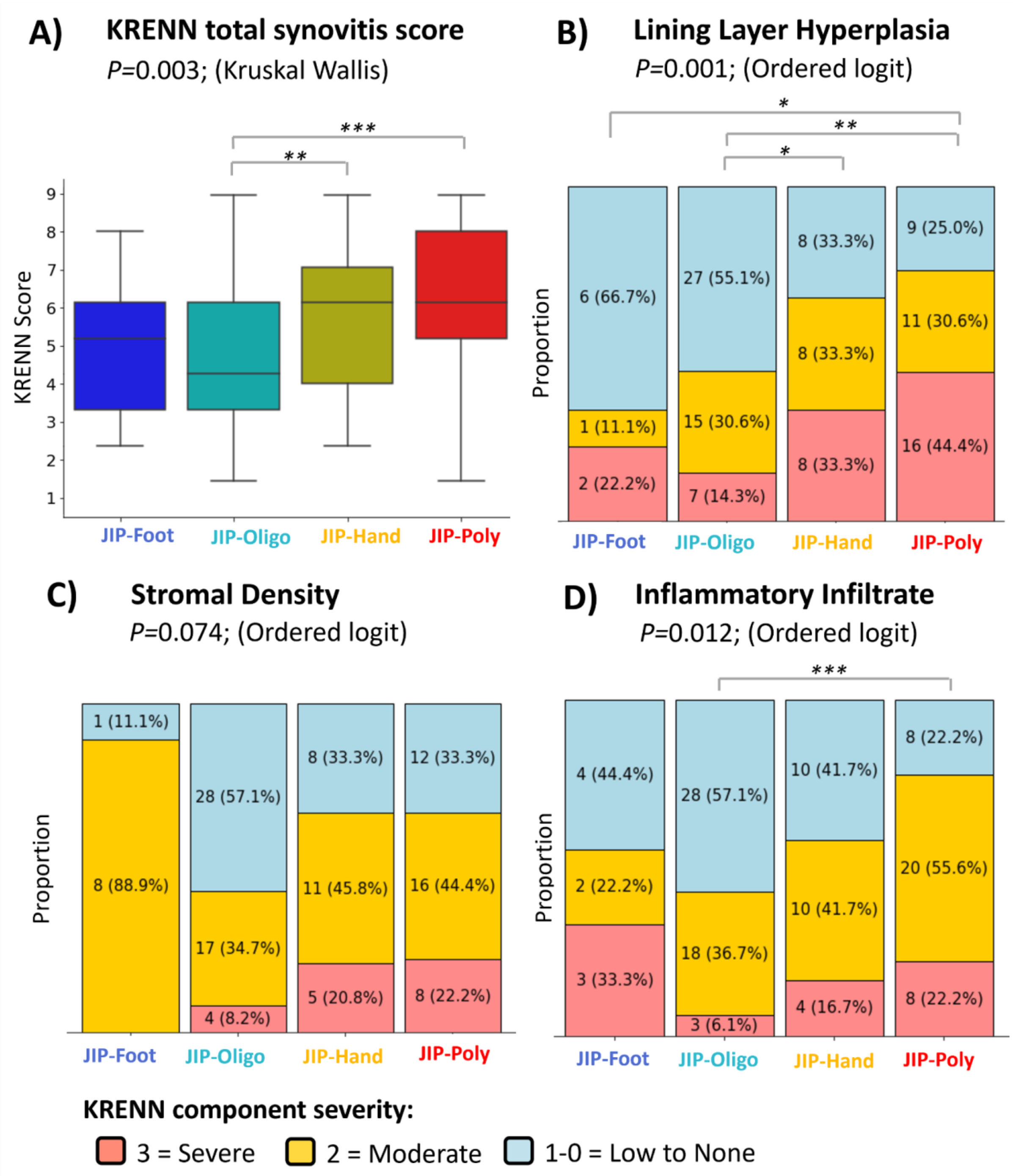

Supplementary Figure 18: Summary of synovitis, using only biopsies taken from JIP-specific joints regions of interest, presented as: a) the total Krenn synovitis score for each cluster, and the individual components: b) lining layer hyperplasia, c) stromal density, and d) inflammatory infiltrate. *Global trends were assessed using the Kruskal-Wallis test for the Krenn synovitis score and an ordered logit model for each subitem grade. These analyses were followed by a post hoc Wald test, with significance levels indicated as *p < 0.05, **p < 0.01, and ***p < 0.001.*

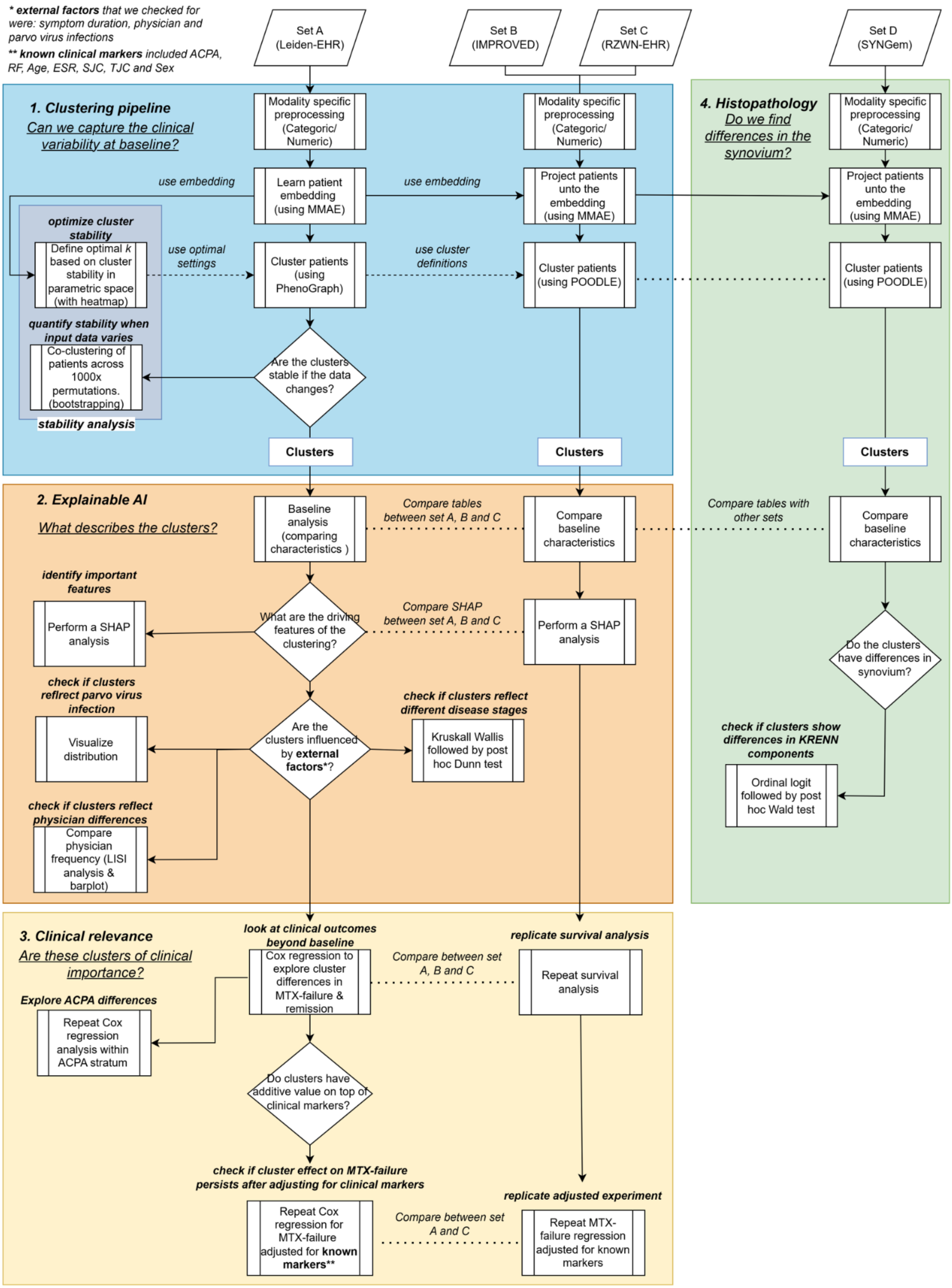
* external factors that we checked for were: symptom duration, physician and parvo virus infections
** known clinical markers included ACPA, RF, Age, ESR, SJC, TJC and Sex
Set A (Leiden-EHR)
Set B (IMPROVED)
Set C (RZWN-EHR)
Set D (SYNGem)
1. Clustering pipeline
Can we capture the clinical variability at baseline?
Modality specific preprocessing (Categoric/ Numeric)
Learn patient embedding (using MMAE)
use embedding
Project patients unto the embedding (using MMAE)
optimize cluster stability
Define optimal k based on cluster stability in parametric space (with heatmap)
use optimal settings
Cluster patients (using PhenoGraph)
use cluster definitions
Cluster patients (using POODLE)
quantify stability when input data varies
Co-clustering of patients across 1000x permutations. (bootstrapping)
Are the clusters stable if the data changes?
stability analysis
Clusters
4. Histopathology
Do we find differences in the synovium?
2. Explainable AI
What describes the clusters?
Baseline analysis (comparing characteristics)
Compare tables between set A, B and C
Compare baseline characteristics
Compare tables with other sets
identify important features
Perform a SHAP analysis
What are the driving features of the clustering?
Compare SHAP between set A, B and C
Do the clusters have differences in synovium?
check if clusters reflrect parvo virus infection
Visualize distribution
Are the clusters influenced by external factors*?
check if clusters reflect different disease stages
Kruskall Wallis followed by post hoc Dunn test
check if clusters show differences in KRENN components
Ordinal logit followed by post hoc Wald test
check if clusters reflect physician differences
Compare physician frequency (LISI analysis & barplot)
3. Clinical relevance
Are these clusters of clinical importance?
look at clinical outcomes beyond baseline
Cox regression to explore cluster differences in MTX-failure & remission
replicate survival analysis
Compare between set A, B and C
Repeat survival analysis
Explore ACPA differences
Repeat Cox regression analysis within ACPA stratum
Do clusters have additive value on top of clinical markers?
check if cluster effect on MTX-failure persists after adjusting for clinical markers
replicate adjusted experiment
Repeat Cox regression for MTX-failure adjusted for known markers**
Compare between set A and C
Repeat MTX-failure regression adjusted for known markers

Supplementary Figure 19: Technical flow highlighting the important methodological steps of our study: 1) Creating clusters, 2) Explainable AI, 3) Clinical relevance, 4) Histopathological analysis. *Where EHR, Electronic health records repository of Leiden University Medical Center (LUMC); AI, Artificial Intelligence; RZWN, hospital data from Reumazorg Zuid West Nederland;*
